# *Ginkgo biloba* Extract (GbE) Stimulates the Hypothalamic Serotonergic System and Attenuates Obesity in Ovariectomized Rats

**DOI:** 10.3389/fphar.2017.00605

**Published:** 2017-09-05

**Authors:** Renata M. Banin, Iracema S. de Andrade, Suzete M. Cerutti, Lila M. Oyama, Mônica M. Telles, Eliane B. Ribeiro

**Affiliations:** ^1^Disciplina de Fisiologia da Nutrição, Departamento de Fisiologia, Universidade Federal de São Paulo São Paulo, Brazil; ^2^Setor de Morfofisiologia e Patologia, Departamento de Ciências Biológicas, Universidade Federal de São Paulo Diadema, Brazil

**Keywords:** ovariectomy, obesity, serotonin, hypothalamus, food intake, *in vivo* microdialysis, *Ginkgo biloba* extract

## Abstract

Menopause is associated with increased risk to develop obesity but the mechanisms involved are not fully understood. We have shown that *Ginkgo biloba* extract (GbE) improved diet-induced obesity. Since GbE might be effective in the treatment of obesity related to menopause, avoiding the side effects of hormone replacement therapy, we investigated the effect of GbE on hypothalamic systems controlling energy homeostasis. Wistar rats were either ovariectomized (OVX) or Sham-operated. After 2 months, either 500 mg.kg^-1^ of GbE or vehicle were administered daily by gavage for 14 days. A subset of animals received an intracerebroventricular (i.c.v.) injection of serotonin (300 μg) or vehicle and food intake was measured after 12 and 24 h. Another subset was submitted to *in vivo* microdialysis and 5-HT levels of the medial hypothalamus were measured by high performance liquid chromatography, before and up to 2 h after the administration of 500 mg.kg^-1^ of GbE. Additional animals were used for quantification of 5-HT_1A_, 5-HT_1B_, 5-HT_2C_, 5-HTT, and pro-opiomelanocortin hypothalamic protein levels by Western blotting. OVX increased food intake and body weight and adiposity while GbE attenuated these alterations. i.c.v. serotonin significantly reduced food intake in Sham, Sham + GbE, and OVX + GbE groups while it failed to do so in the OVX group. In the OVX rats, GbE stimulated 5-HT microdialysate levels while it reduced hypothalamic 5-HTT protein levels. The results indicate that GbE improved the ovariectomy-induced resistance to serotonin hypophagia, at least in part through stimulation of the hypothalamic serotonergic activity. Since body weight gain is one of the most important consequences of menopause, the stimulation of the serotonergic transmission by GbE may represent a potential alternative therapy for menopause-related obesity.

## Introduction

Menopause is a physiological condition characterized by the loss of ovarian function (Dalal and Agarwal, 2015). Hypoestrogenism has been identified as the main factor of menopause-associated alterations, which include obesity, decreased energy expenditure, vasomotor symptoms, insomnia, and psychological disturbances, all affecting negatively the quality of life (Al-Safi and Santoro, 2014; Jenabi et al., 2015).

Estrogens are known to play an important role in the homeostatic control of food intake and body weight (Eckel, 2011; Asarian and Geary, 2013). During the estrous phase, female rats exhibited a decrease in the size of spontaneous meals, a pattern similar to that observed in women, which presented daily food intake alterations during the hormonal fluctuations of the ovarian cycle. On the other hand, estradiol replacement attenuated ovariectomy-induced binge eating and high body weight in rats (Yu et al., 2011; Baker and Runfola, 2016).

A role of estrogens as modulators of the central serotonergic activity has been demonstrated, but with both stimulatory and inhibitory effects on the system. Indeed, they reportedly increased the expression of tryptophan hydroxylase and of serotonergic receptors and auto-receptors (Bethea et al., 2002, 2016; Hiroi and Neumaier, 2009; Barth et al., 2015).

Central serotonin (5-HT) plays a pivotal role in the control of energy homeostasis due to its anorexigenic action, exerted mainly via the receptor subtypes 5-HT_1B_ and 5-HT_2C_ (Huang et al., 2004). Moreover, increased 5-HT availability, as evoked by 5-HT reuptake inhibitors, induces hypophagia in both humans and animals (Huang et al., 2004; Olivier and van Oorschot, 2005). However, studies about the central activity of 5-HT in a situation of lack of ovarian hormones are very scarce.

*Ginkgo biloba* is one of the herbal medicines most used worldwide (Diamond and Bailey, 2013). *G. biloba* 761 standardized extract (GbE 761), made from its dried green leaves, is one of the best extracts related to therapeutic purposes. It has been described that *G. biloba* has beneficial effects on neurological disturbances, including memory loss, dementia, mood fluctuations, psychomotor symptoms, and psychiatric disorders such as schizophrenia and Alzheimer’s disease (Diamond and Bailey, 2013; Montes et al., 2015). We have recently demonstrated that GbE administration to diet-induced obese rats led to significant decrements of food intake, body weight gain and visceral adiposity, suggesting its potential as a weight management therapy (Banin et al., 2014; Hirata et al., 2015).

There are few studies examining the effect of GbE on serotonergic pathways. In a review about its neuroprotective effects, GbE has been reported to prevent the aging-induced reductions in 5-HT levels and 5-HT_1A_ receptors density in the rat cerebral cortex. Furthermore, GbE 761 stimulated 5-HT uptake by cortical synaptosomes of mice, an action attributed to its flavonoid component quercetin (Ahlemeyer and Krieglstein, 2003). GbE 761 has recently been shown to inhibit reuptake transporters of norepinephrine, serotonin, and dopamine, as well as to decrease monoamine oxidase (MAO) activity (Fehske et al., 2009). Interestingly, the use of GbE and other herbal medicines, as alternative or complementary therapy, by post-menopausal women has been shown improve mood, hot flashes, and cognitive symptoms (Kam et al., 2002; Clement et al., 2011). However, there is no data about beneficial effects of GbE on the control of food intake and body weight gain related to menopause.

The present study aimed at investigating, in an ovariectomized (OVX) rat model, whether the lack of ovarian hormones affects aspects of the central serotonergic system related to energy balance and whether GbE therapy is able to modify these effects.

## Materials and Methods

### Animals and Ovariectomy Surgery

All procedures were approved by the Ethics Committee on Animal Research of the Universidade Federal de São Paulo (process number: CEP 0024/13), which follows the guidelines of National Institutes of Health guide for the care and use of Laboratory animals (NIH Publications No. 8023, revised 1978). Throughout the experimental period, the female Wistar rats were maintained under controlled temperature (23 ± 1°C) and lighting conditions (lights on from 6 a.m. to 6 p.m.), with free access to water and balanced diet (Nuvilab^®^, Brazil, 2.79 kcal.g^-1^).

OVX (*n* = 49) or Sham (*n* = 48) were performed by the dorsal approach in 2-month-old female rats anesthetized with ketamine/xylazine (66.6/13.3 mg.kg^-1^, i.p.) and treated with an oral dose (25 mg.kg^-1^ body weight) of ibuprofen. After the surgery, a subcutaneous dose of 300,000 U.kg^-1^ body weight of penicillin (1,200,000U benzathine benzylpenicillin Bepeben^®^) was administered. An analgesic reinforcement was carried out 12, 24, and 36 h after the surgery. In all animals, ovariectomy was confirmed at the end of the experimental period, by the atrophy of the uteri, contrasting with the false-OVX animals.

Both body weight and daily food intake were measured once weekly for 8 weeks since the surgeries and then the phytotherapy treatment was initiated. Food efficiency was calculated by the ratio of body weight gain (g)/food ingestion (g) (de Sá et al., 2016) during the 8-week period after ovariectomy.

### Phytotherapy Treatment

GbE was obtained from Huacheng Biotech Inc. (China). The composition of GbE included flavone glycosides (25.21%), terpenoids (6.62%), ginkgolides A, B, C (3.09%), and bilobalides (2.73%).

**FIGURE 1:**
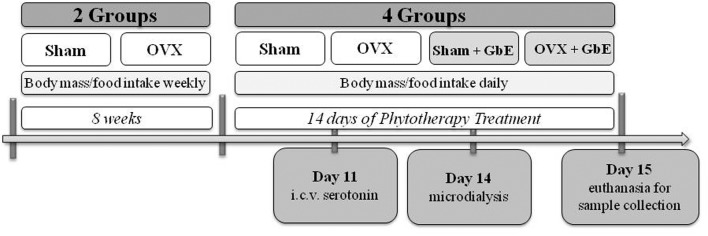
Timeline of the experimental design.

Two months after ovariectomy, the Sham and OVX rats were randomly divided into four groups, treated with either vehicle or GbE: Sham (*n* = 24), Sham + GbE (*n* = 24), OVX (*n* = 24), and OVX + GbE (*n* = 25). The treatment consisted of a gavage with 500 mg.kg^-1^ GbE diluted in 1.5 mL 0.9% saline (groups Sham + GbE and OVX + GbE) or 1.5 mL of vehicle (groups Sham and OVX) and was performed once daily for 14 days. During the treatment, body weight and food intake were measured daily and cumulative food intake was calculated as the sum of the daily dietary intakes of the whole treatment period. A schematic timeline of major events involving the experimental groups is shown in **Figure 1**.

### Stereotaxic Surgery

At the 7th day of phytotherapy treatment, the rats were anesthetized with ketamine/xylazine (66.6/13.3 mg.kg^-1^; i.p.) and, after the administration of an oral dose (25 mg.kg^-1^ of body weight) of ibuprofen, they were submitted to stereotaxic surgery. For the intracerebroventricular (i.c.v.) serotonin injection, a subset of animals received a 21 gauge cannula (15 mm length) aimed at the left lateral ventricle [from bregma: anteroposterior (AP) = -0.9 mm; mediolateral (ML) = +1.6 mm; dorsoventral (DV) = -2.5 mm]. For the microdialysis study, a subset of animal received a 20 gauge cannula (20 mm length) aimed at the right ventromedial hypothalamus (from bregma: AP = -2.6 mm; ML = -0.6 mm; DV = -7.8 mm). The cannulas were secured to the skull with screws and dental cement. Immediately after the surgery, a subcutaneous dose of penicillin (300,000 U.kg^-1^, Bepeben^®^) was administered. The rats were then placed in individual cages with free access to water and food and continued to receive the GbE treatment for the following 7 days. Food intake and body weight were measured daily to check for the post-surgical recovery.

### Food Intake Measurement after Intracerebroventricular Serotonin

After 3 days of the lateral ventricle cannulation, the animals were fasted for 6 h and received an i.c.v. injection of 5.0 μL of vehicle (artificial cerebrospinal fluid: 145 mM NaCl, 2.7 mM KCl; 1.0 mM MgCl_2_; 1.2 mM CaCl_2_; 2.0 mM Na_2_HPO_4_; pH 7.4) or 5.0 μL of vehicle containing 300 μg of 5-HT (H9523, Sigma-Aldrich, United States). The injections were performed immediately before lights out. After the injection, the animals were returned to their individual cages and a known amount of chow was offered. Food consumption was determined by weighting the remaining chow after 12 and 24 h of the injection. Each animal was injected twice, receiving vehicle or serotonin, on separate days, with an interval of 2 days between the experiments. The rats were randomly divided, so that half of the animals received vehicle as first injection and the other half received serotonin as first injection. The correct cannula positioning was evaluated by the dipsogenic effect of 20 ng of angiotensin II (i.c.v.).

### *In Vivo* Microdialysis

On the 13th day of the phytotherapy treatment, around 5 p.m., food was removed and the microdialysis probe was inserted into the guide cannula. The probes were custom-constructed, as previously described, with 2.0 mm of effective membrane length and 13,000 Da cut-off (Mori et al., 1999; Pôrto et al., 2009). The probe inlet was connected to a micro-infusion pump (CMA-Harvard Apparatus, Kista, Stockholm, Sweden) and the animals were connected to a swivel system (CMA Microdialysis^®^) allowing continuous perfusion with artificial cerebrospinal fluid. Overnight infusion flow rate was 1.0 μL.min^-1^. In the next day, around 8 a.m., flow rate was adjusted to 2.5 μL.min^-1^. After allowing 60 min for stabilization, collection of 20-min dialysate samples was started. Samples were collected into 10 μL of 0.5 M perchloric acid and immediately injected into a high performance liquid chromatography (HPLC) system (Mori et al., 1999; Telles et al., 2003). All dialysate collections were performed between 9 and 12 a.m. Baseline samples were collected until 5-HT levels were stable. Then, the Sham + GbE and the OVX + GbE animals received 500 mg.kg^-1^ of GbE by gavage while the Sham and OVX groups received 1.5 mL of vehicle. After the gavage procedure, six additional 20-min. microdialysate samples were collected.

### HPLC Analysis

Dialysate levels of 5-HT were measured by HPLC with electrochemical detection. The system (ESA, Inc., Chelmsford, United States) consisted of a pump with two PEEK pulse dampers in series, a 50 μL Rheodyne PEEK sample loop, a 3 μM MD150 C18 reverse phase column, a model 5020 guard cell with 300 mV potential, a model 5014A analytical cell set a -175 and 150 mV in the first and second electrodes, respectively, and a model 5200A detector. The mobile phase consisted of 75 mM sodium phosphate, 1.5 mM octanesulfonic acid, 50 μM EDTA, 150 μL triethylamine.L^-1^, 20% acetonitrile, pH 3.0 at a flow rate of 0.6 mL.min^-1^ (Mori et al., 1999; Guimarães et al., 2002; Telles et al., 2003).

### Histological Analysis and Weighing of Visceral Adipose Tissue Depots

One day after the microdialysis experiment, the rats were deeply anesthetized with thiopental (80 mg.kg^-1^; i.p.), the abdominal cavity was opened and the retroperitoneal and mesenteric fat pads were removed and weighed. The animals were then perfused intracardially with PBS solution followed by 4% paraformaldehyde dissolved in PBS. Brains were removed and kept in 4% paraformaldehyde for 1 week and then in 30% sucrose solution for 4 days. The 45 μM sections were obtained in a cryostat, stained with cresyl violet and examined under an optical microscope for confirmation of the positioning of the microdialysis membranes in the ventromedial hypothalamus region. We considered only the results obtained in those animals in which the microdialysis membrane was correctly positioned.

### Western Blotting Analysis

Additional rats were killed after an 8 h-fasting. The hypothalami were removed and homogenized in 0.8 mL of solubilization buffer (100 mM Tris, pH 7.5, 0.1 mg.mL^-1^ aprotinin, 2 mM phenylmethylsulfonyl fluoride, 10 mM sodium orthovanadate, 100 mM sodium fluoride, 10 mM sodium pyrophosphate, and 10 mM EDTA). Triton X-100 was added to a final concentration of 10%. Samples were centrifuged at 16,000 *g* for 40 min and 50 μg of protein were resolved in SDS–polyacrylamide gel electrophoresis, transferred to nitrocellulose membranes and incubated with primary antibodies against the serotonergic receptors 5-HT_1A_ (anti-5-HT_1A_, ab79230)_,_ 5-HT_1B_ (anti-5-HT_1B_, ab13896), and 5-HT_2C_ (anti-5-HT_2C_, ab133570), the serotonin transporter—5-HTT (anti-5-HTT, ab172884), and the anorexigenic mediator pro-opiomelanocortin (POMC; anti-POMC, ab180766). The blots were incubated with peroxidase-conjugated secondary antibodies and specific bands were detected by chemiluminescence (GE Healthcare Bio-Sciences, Pittsburgh, PA, United States).

For evaluation of protein loading, all membranes were stripped and re-blotted with anti-β-tubulin (Ab#2146) primary antibody. Band intensities were quantified by optical densitometry (Scion Image software, Scion Corporation, Frederick, MD, United States). The measured density of all proteins where then corrected to the density of β-tubulin levels. The results are expressed as percentage of the intensity measured in the Sham group. The whole pictures of Western blotting analysis are provided in the Supplementary Data Sheet 1.

### Statistical Analysis

Data are expressed as mean ± SEM. The PASW Statistics version 21 software (SPSS Inc., United States) was adopted in all analysis with the level of statistical significance set at *p* ≤ 0.05.

Comparisons of body mass and relative food intake within Sham and OVX groups were performed by one-way ANOVA with repeated measures. Comparisons of body mass, food intake, body weight gain, and food efficiency between Sham and GbE groups were carried out by Student’s *t*-test for independent samples. Comparisons of body weight gain, relative food intake, visceral adipose tissues depots, and uterus mass among all groups were analyzed by two-way ANOVA followed by Tukey HSD test adopting OVX and GbE as the fixed factors. The effect of acute i.c.v. 5-HT on food intake in each group was assessed by the paired Student’s *t*-test.

5-HT microdialysate levels are expressed as percentage of the mean baseline level. For comparisons among the samples collected throughout the experimental period in the same group, one-way ANOVA followed by Tukey HSD test was performed. The area under the curve (AUC) relating serotonin levels to time after GbE or vehicle administration was calculated by the trapezoidal rule. The comparisons of microdialysate 5-HT levels among the four groups in each time point, serotonin AUC, and Western blotting data were analyzed by two-way ANOVA followed by Tukey HSD test considering OVX and GbE as main factors. Detailed tables of statistical analysis are available in the Supplementary Data Sheet 2.

## Results

### Body Weight and Food Intake during the 8 Weeks after Ovariectomy

**FIGURE 2:**
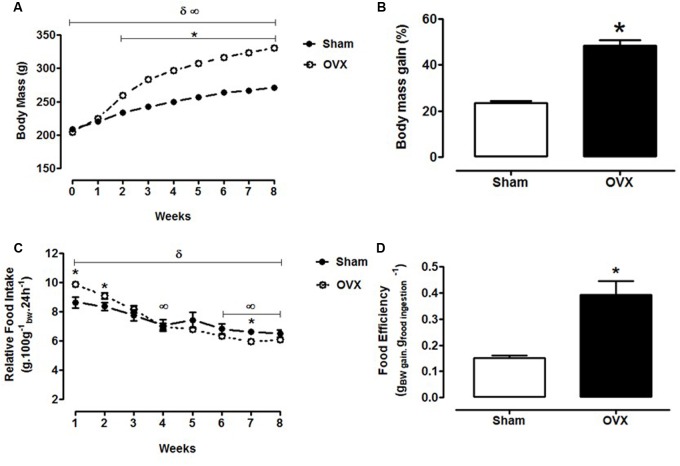
Body mass and food intake during the 8 weeks after ovariectomy or false-ovariectomy. **(A)** Body mass; **(B)** body mass gain (% of initial body mass); **(C)** relative food intake (g.100 g^-1^_bw_.24 h^-1^); and **(D)** food efficiency (g.kcal^-1^) of Sham (*n* = 48) and OVX rats (*n* = 49). ^∞^*p* ≤ 0.05 *vs.* week 0 within the Sham group; ^δ^*p* ≤ 0.05 *vs.* week 0 within the OVX group; ^∗^*p* ≤ 0.05 *vs.* Sham.

Both Sham [*F*_(1,47)_ = 3928.55; *p* < 0.0001] and OVX [*F*_(1,48)_ = 7808.63; *p* < 0.0001] rats showed a progressive elevation of their body masses, from week 0 (initial body mass) to the end of week 8, as it can be observed in **Figure 2A**. In comparison to the Sham group, the OVX rats presented significantly higher body mass from the 2nd to the 8th week after ovariectomy (week 2: 11%; week 3: 17%; weeks 4 and 5: 19%; week 6: 20%; week 7: 21%; and week 8: 22%, *p* < 0.0001). The total body mass gain of the OVX group was 107% higher than that of the Sham group (*p* < 0.0001), as it can be verified in **Figure 2B**.

**Figure 2C** shows that the Sham rats exhibited a significant reduction of the relative food intake at the 4th (*p* = 0.015), 6th (*p* = 0.002), 7th (*p* = 0.001), and 8th weeks (*p* < 0.0001), in relation to the 1st week of evaluation [*F*_(1,10)_ = 1577.10; *p* < 0.0001]. In the OVX group, the relative food intake decreased significantly [*F*_(1,8)_ = 4712.82; *p* < 0.0001] since the 2nd week (week 2: -7%, *p* = 0.044; week 3: -17%, *p* = 0.001; week 4: -28%, *p* < 0.0001; week 5: -29%, *p* < 0.0001; week 6: -34%, *p* < 0.0001; week 7: -36%, *p* < 0.0001; and week 8: -34%, *p* < 0.0001). In comparison to the Sham rats, the OVX group presented a significantly higher food intake at the 1st (14%, *p* = 0.006) and 2nd weeks (9%, *p* = 0.022). However, this group presented a significant reduction in food intake in the 7th week in relation to Sham group (-10%, *p* = 0.009). By the end of the 8th week, food intake was similar between the groups. In addition, **Figure 2D** shows that the OVX group presented a 160% increase of food efficiency (*p* < 0.0001) in comparison to the Sham group.

### Body and Adipose Tissue Mass and Food Intake in Response to 14 Days of GbE Treatment

**FIGURE 3:**
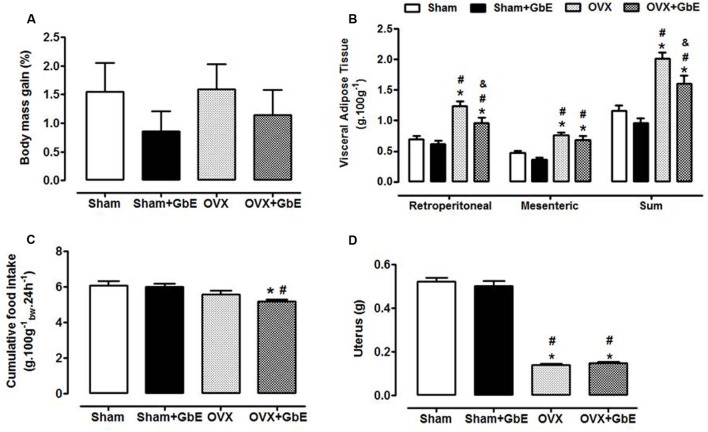
Body mass gain and cumulative food intake during the 14 days of GbE therapy, and final fat depots and uterus mass. **(A)** Body mass gain (% of initial body mass) of Sham (*n* = 13), Sham + GbE (*n* = 12), OVX (*n* = 14), and OVX + GbE (*n* = 11) groups; **(B)** visceral adipose tissue depots mass (g.100 g^-1^) of Sham (*n* = 21–24), Sham + GbE (*n* = 21–23), OVX (*n* = 18–25), and OVX + GbE (*n* = 18–23) groups; **(C)** cumulative food intake (g.100 g^-1^_bw_.24 h^-1^) of Sham (*n* = 13), Sham + GbE (*n* = 12), OVX (*n* = 14), and OVX + GbE (*n* = 11) groups; **(D)** uterus mass (g) Sham (*n* = 21–24), Sham + GbE (*n* = 21–23), OVX (*n* = 18–25), and OVX + GbE (*n* = 18–23) groups. ^∗^*p* ≤ 0.05 *vs.* Sham; ^#^*p* ≤ 0.05 *vs.* Sham + GbE; ^&^*p* ≤ 0.05 *vs.* OVX.

Body mass gain during the 14 days of GbE treatment failed to show significant differences among the groups [*F*_(3,51)_ = 0.506, *p* = 0.680, **Figure 3A**]. Ovariectomy increased the mass of the retroperitoneal [*F*_(3,79)_ = 45.66, *p* < 0.0001] and the mesenteric [*F*_(3,79)_ = 49.44, *p* < 0.0001] fat depots and of the sum of these visceral fat depots [*F*_(3,79)_ = 54.96, *p* < 0.0001], in comparison to Sham and the Sham + GbE groups. GbE attenuated these adiposity measures, as the OVX + GbE rats showed a 23% reduction (*p* = 0.027) of retroperitoneal fat pad mass [*F*_(3,79)_ = 7.47, *p* = 0.008] and a 20% reduction (*p* = 0.035) of visceral fat pads sum [*F*_(3,79)_ = 54.96, *p* < 0.0001], in relation to the OVX rats (**Figure 3B**).

The cumulative food intake [*F*_(3,22)_ = 12.30, *p* = 0.003] was lower in OVX + GbE than in Sham (-15%, *p* = 0.019) and Sham + GbE (-14%, *p* = 0.023) groups (**Figure 3C**). As expected, the lack of ovarian hormones promoted a significant atrophy of the uteri [*F*_(3,95)_ = 584.46; *p* < 0.0001], as compared to both Sham and Sham + GbE groups (**Figure 3D**).

### Food Intake in Response to Intracerebroventricular Serotonin

**FIGURE 4:**
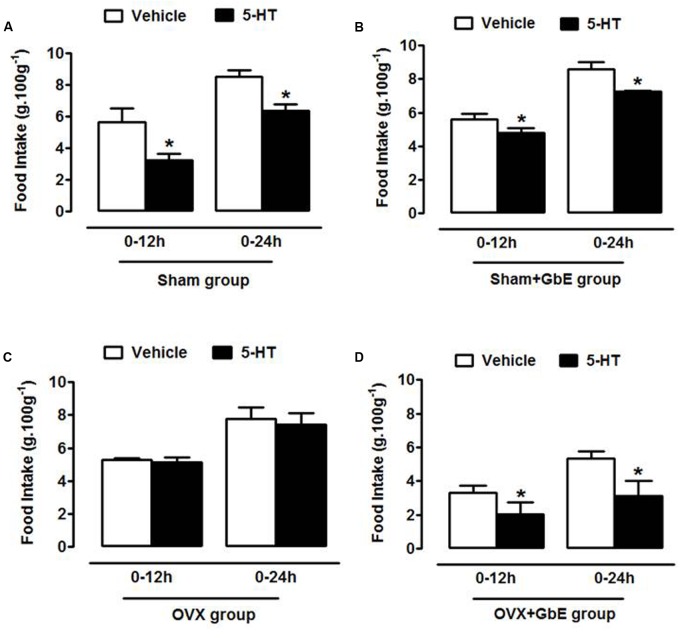
Food intake in response to intracerebroventricular serotonin. Relative food intake of **(A)** Sham (*n* = 4), **(B)** Sham + GbE (*n* = 4), **(C)** OVX (*n* = 4), and **(D)** OVX + GbE (*n* = 5) in the 12 and 24 h periods after the intracerebroventricular injection of either vehicle or 300 mg 5-HT. ^∗^*p* ≤ 0.05 *vs.* vehicle.

Serotonin infusion significantly reduced food intake in relation to vehicle infusion in the Sham (-42%, *p* = 0.0017 and -25%, *p* = 0.0010; **Figure 4A**) and Sham + GbE (-14%, *p* = 0.024 and -15%, *p* = 0.040; **Figure 4B**) groups. Serotonin-induced hypophagia was abolished in the OVX group (**Figure 4C**) and was restored by the GbE treatment (-39%, *p* = 0.047 and -42%, *p* = 0.037; **Figure 4D**).

### Effect of GbE on Serotonin Levels in Microdialysates of the Medial Hypothalamus

Basal extracellular levels of serotonin were similar among Sham (1.05 ± 0.22 pg.50 μL^-1^, *n* = 6–7), Sham + GbE (1.84 ± 0.35 pg.50 μL^-1^, *n* = 5–6), OVX (1.40 ± 0.38 pg.50 μL^-1^, *n* = 5–10) and OVX + GbE (1.11 ± 0.20 pg.50 μL^-1^, *n* = 7–10); [*F*_(3,30)_ = 1.172, *p* = 0.339] groups.

**FIGURE 5:**
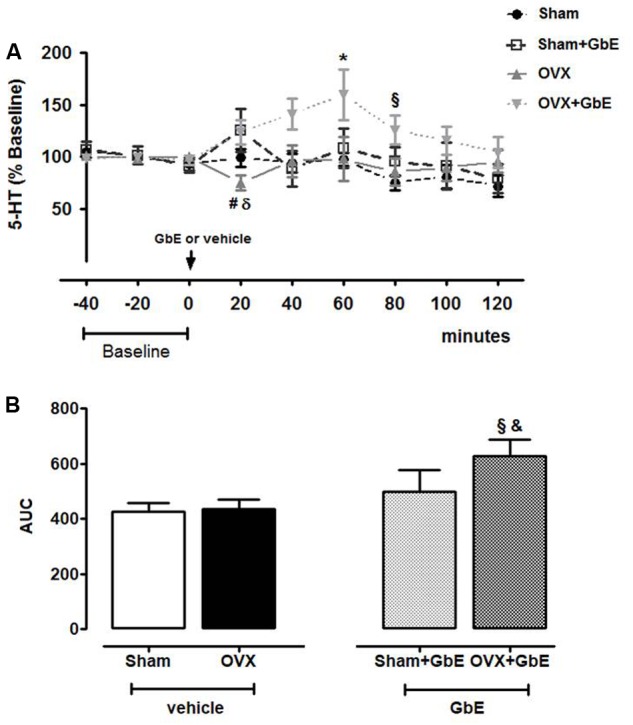
Medial hypothalamic serotonin extracellular levels as measured by *in vivo* microdialysis. **(A)** 5-HT levels (% baseline) in 20-min VMH microdialysate samples collected before (baseline –40 to 0 min) and up to 120 min after gavage with either vehicle or 500 mg.kg^-1^ of GbE and **(B)** area under the curve (AUC) of 5-HT extracellular levels during the 120 min of microdialysate collection after gavage of Sham (*n* = 6–7), Sham + GbE (*n* = 5–6), OVX (*n* = 5–10), and OVX + GbE (*n* = 7–10). ^∗^*p* ≤ 0.05 *vs.* baseline; ^x^ *p* ≤ 0.05 *vs.* Sham; ^#^*p* ≤ 0.05 *vs.* Sham + GbE; ^&^*p* < 0.05 *vs.* OVX; ^δ^*p* ≤ 0.05 *vs.* OVX + GbE.

The gavage with vehicle failed to significantly affect 5-HT extracellular levels in both the Sham [*F*_(8,58)_ = 2.427, *p* = 0.072] and the OVX groups [*F*_(8,68)_ = 0.7189, *p* = 0.674]. The injection of GbE failed to significantly alter 5-HT microdialysate levels in the Sham + GbE group [*F*_(8,52)_ = 0.7869, *p* = 0.617]. On the other hand, it caused a significant elevation of 5-HT levels in the OVX + GbE group [*F*_(8,77)_ = 2.675, *p* = 0.013]. The levels were significantly elevated from baseline at the sample collected 60–80 min after the acute dose of GbE (**Figure 5A**).

The comparison among the groups at each time point showed that GbE promoted a significant increment of 5-HT levels [*F*_(3,29)_ = 9.255, *p* = 0.005] in both Sham + GbE and OVX + GbE rats, in the 20–40 min sample (67%, *p* = 0.042; 64%, *p* = 0.033, respectively) in comparison to the OVX group. GbE also increased 5-HT level of the OVX + GbE group in relation to that of the Sham group at the 80–100 min sample [67%, *p* = 0.029; *F*_(3,27)_ = 5.292, *p* = 0.031; **Figure 5A**].

The AUC relating serotonin levels to time after GbE administration was higher in OVX + GbE group [*F*_(3,31)_ = 6.425, *p* = 0.017] in comparison to the Sham (47%, *p* = 0.046) and the OVX group (45%, *p* = 0.040) (**Figure 5B**).

### Effects of Ovariectomy and GbE on Hypothalamic Protein Expression

**FIGURE 6:**
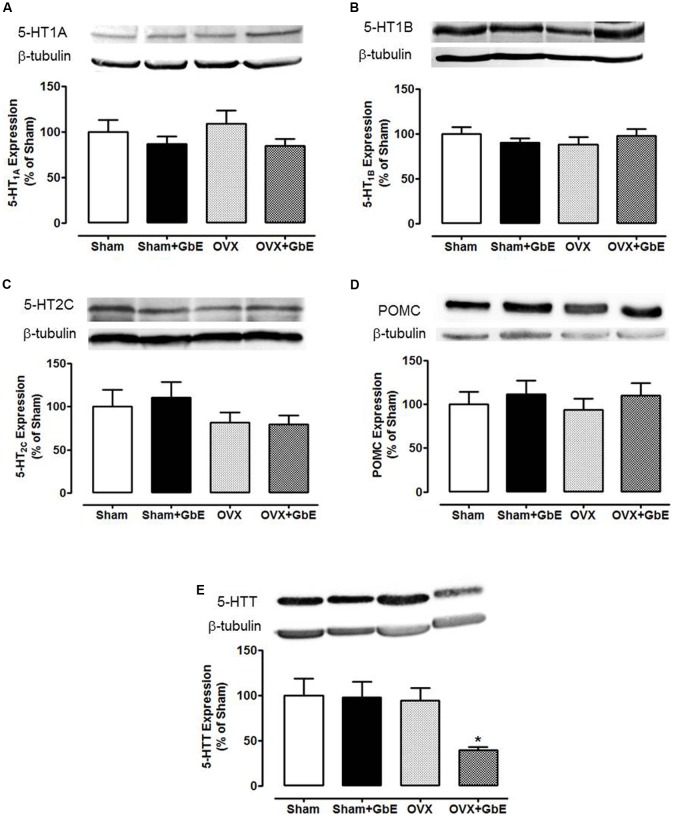
Western blotting analysis of the hypothalamic expression of the proteins serotonin receptors 1A, 1B, and 2C, serotonin transporter (5-HTT), and POMC. **(A)** 5-HT_1A_; **(B)** 5-HT_1B_; **(C)** 5-HT_2C_; **(D)** POMC; and **(E)** 5-HTT expression, in % of Sham group expression. Sham (*n* = 12–13), Sham + GbE (*n* = 11–12), OVX (*n* = 12–14), and OVX + GbE (*n* = 10–11). ^∗^*p* < 0.05 *vs.* Sham.

No differences were observed among the groups in protein levels of 5-HT_1A_ [*F*_(3,48)_ = 0.928, *p* = 0.435], 5-HT_1B_ [*F*_(3,49)_ = 0.589, *p* = 0.625], 5-HT_2C_ [*F*_(3,49)_ = 0.865, *p* = 0.465], and POMC [*F*_(3,45)_ = 0.371, *p* = 0.774] (**Figures 6A–D**). However, there was a significant 60% reduction (*p* = 0.043) in the protein levels of the 5-HTT (**Figure 6E**) [*F*_(3,45)_ = 3.321, *p* = 0.029] of OVX + GbE rats in relation to that of the Sham group. Additionally, that group also showed a trend to elevated levels in relation to the Sham + GbE (*p* = 0.054) and OVX (*p* = 0.075) groups.

## Discussion

It is well recognized that menopause is associated with excess body mass gain and visceral fat deposition (MacLean et al., 2010; Asarian and Geary, 2013; Dalal and Agarwal, 2015). In the present study, ovariectomy induced increased body mass since the 2nd week, leading to a twofold total increase by the 8th week. By this time, visceral adipose depots were augmented, a condition known to be linked with the development of chronic diseases such as insulin resistance, diabetes, cardiovascular disorders, and cancer (Stachowiak et al., 2015).

The daily food intake of the OVX rats showed a transient increase, in the 1st and 2nd weeks, but was normalized by the 8th week. This pattern of feeding agrees with a previous report in OVX rats (Ferreira et al., 2012) and may be attributed to a compensatory mechanism to control energy balance after a period of hyperphagia (Woods, 2013). However, it is important to emphasize that, although both the Sham and the OVX rats exhibited a progressive decrease of food intake over the 8 weeks, the decrement was more pronounced in the OVX group, showing that the excess weight gain did not rely on excess energy consumption. This can be explained, at least in part, by the increased food efficiency shown by the OVX rats, in accordance with a previous report on the consequences of the absence of ovarian hormones (MacLean et al., 2010).

Estrogen replacement has been shown to attenuate obesity in both OVX animals (Yu et al., 2011; Ibrahim et al., 2015) and in post-menopausal women (Butera, 2010). Estrogens regulate food intake by reducing the activity of orexigenic factors, such as ghrelin and neuropeptide Y (NPY), as well as by stimulating anorexigenic signals, such as cholecystokinin, brain-derived neurotrophic factor and the serotonin pathway (Bethea et al., 2002, 2016; Butera, 2010; Zhu et al., 2013; Grochans et al., 2014).

Hormone replacement therapy (HRT) remains the most used treatment for ameliorating menopause-related symptoms, as it presents positive effects on food intake control and body weight gain. Nevertheless, as HRT may lead to important side effects such as breast cancer, heart stroke, and thrombosis, this therapy must be well evaluated and constantly monitored (Pines and Shapiro, 2015). In this context, the development of alternative treatments for menopause management is highly desirable.

Previous studies from our laboratory have demonstrated that GbE was efficient in reducing food intake, body weight gain, and visceral adiposity in diet-induced obese male rats (Banin et al., 2014; Hirata et al., 2015). Due to this positive effect of GbE on energy homeostasis, the present study was aimed at evaluating if this herbal medicine would attenuate the effects of ovarian hormones absence on energy homeostasis and, if so, whether the hypothalamic serotonin system played a role. The results showed that GbE did reduce spontaneous feeding and attenuated body adiposity of OVX rats during the 2-week treatment. Although few studies have addressed the mechanisms involved in GbE-induced hypophagia, a positive effect of GbE on components of serotonergic signaling pathway has been documented (Huguet et al., 1994; Bolaños-Jiménez et al., 1995; Sloley et al., 2000).

Since exogenous 5-HT and agents that enhance synaptic availability of endogenous 5-HT are known to suppress feeding (Leibowitz and Alexander, 1998), in the present work food intake was assessed after an i.c.v. injection of 5-HT. The results demonstrated that OVX rats failed to show hypophagia in response to an acute central infusion of serotonin. This finding agrees with the demonstration of impairment of fenfluramine-induced hypophagia in OVX rats in comparison to estradiol-replaced OVX animals (Rivera and Eckel, 2005). It has been previously reported that fluoxetine decreased appetite and body mass index of post-menopausal women after 2–3 months of treatment but these effects were no longer observed after 6 months of therapy (Chojnacki et al., 2015).

Importantly, GbE improved serotonin effectiveness in the OVX animals, as seen by the restored feeding response to the neurotransmitter in the OVX + GbE group. Along with the observation of the low food intake presented by OVX + GbE rats during the 14-day treatment, this finding allows the suggestion that GbE-induced hypophagia relied, at least in part, on serotonergic mechanisms.

Indeed, the microdialysis experiments evidenced a potent effect of GbE on the serotonergic system of the OVX rats, as the treatment significantly stimulated 5-HT microdialysate levels. This agrees with the demonstration that GbE 761 increased 5-HT uptake by mice cortical synaptosomes, an effect attributed to the flavonoid compounds of GbE (Ramassamy et al., 1992). On the other hand, it contrasts with the observation of a previous report in which the authors did not observe any effect of GbE on the pre-frontal cortex extracellular 5-HT levels of rats (Yoshitake et al., 2010).

Serotonin hypophagia has been attributed to its interaction with receptors 5-HT_1A_, 5-HT_1B_, and 5-HT_2C_ (Huang et al., 2004). Pretreatment with 5-HT_1A_ antagonists impaired the hypophagia induced by fluoxetine administration in the pre-limbic cortex of fasted rats (Stanquini et al., 2015). Acting on 5-HT_2C_ receptors, 5-HT has been shown to induce the cleavage of POMC into the active anorexigenic factor α-melanocyte-stimulating hormone (α-MSH). Acting on 5-HT_1B_, serotonin reportedly inhibited the orexigenic mediators NPY and agouti-related protein (AgRP) and activated the anorexigenic peptides α-MSH and cocaine- and amphetamine-regulated transcript (CART) (Pratt et al., 2016). Inhibition of the 5-HTT decreased food intake and body weight in rats and humans (Huang et al., 2004; Olivier and van Oorschot, 2005).

Available data addressing effects of GbE in the modulation of serotonergic receptors involved in energy homeostasis are very scarce. In the rat brain, GbE has been shown to prevent the stress-induced 5-HT_1A_ desensitization in the hippocampus and to reverse the aging-induced decrement of cortical 5-HT_1A_ density (Huguet et al., 1994; Bolaños-Jiménez et al., 1995). A neuroprotective ability of GbE has been proposed based both on the ability of the phytoestrogen kaempferol, isolated from GbE leaves, to reduce MAO-A/B activity in rat brain cells in culture, and on its antioxidant effect in a lipid-peroxidation assay (Sloley et al., 2000). A better understanding of the central targets of GbE is relevant for the proposal of beneficial effects of this herbal medicine on body weight gain, particularly after menopause.

In the present study, GbE failed to affect hypothalamic levels of 5-HT_1A_, 5-HT_1B_, 5-HT_2C_, and POMC. On the other hand, it induced a 60% significant decrement of hypothalamic 5-HTT density, only in the OVX rats. It is, thus, possible to suggest that GbE increased serotonin effectiveness through reuptake inhibition, what was probably relevant for the reduction of food intake and stimulation of 5-HT microdialysate levels induced by GbE. Differently from its action on the OVX rats, GbE had no effect on the hypothalamic 5-HTT levels in the Sham-OVX group, a finding consistent with its lack of effect on feeding and microdialysate serotonin levels in this group.

As mentioned above, estrogens’ effects on the serotonergic system are complex. Although the majority of the studies have pointed to an overall stimulatory effect of estrogens, inhibitory consequences have also been described (Barth et al., 2015). Low estrogen levels have been associated with reduced 5-HT concentration in some brain areas, such as cortex and striatum (Hao et al., 2011). Furthermore, OVX rats reportedly presented a marked reduction in the number of serotonin-positive neurons in the dorsal raphe nuclei. This reduction was reversed by either estrogen or remifemin, a herbal medicine containing triterpene glycosides and phenolic substances, used for the relief of menopausal symptoms (Wang et al., 2016).

The present results also indicated a stimulatory action, as evidenced by the absence of serotonin hypophagia and the slightly decreased microdialysate levels in the OVX group. A recent study in mice has shown an inverse relationship between estrogen and serotonergic activity, as 5-HT striatal microdialysate levels were found to be lower in the high estrogen than in the low estrogen phase of the ovarian cycle. Importantly, this effect was linked to inhibition of 5-HTT expression (Yang et al., 2015), similarly to the present observation concerning the effect of GbE in the OVX rats. These considerations raise the suggestion that the positive role of GbE on serotonergic regulation may rely on mechanisms similar to those targeted by estrogens. This agrees with the absence of GbE’s effects on the Sham-OVX animals.

In human breast cancer cells, both estrogenic and antiestrogenic actions of GbE have been demonstrated, in a biphasic manner dependent on both estrogen and GbE concentrations. The antiestrogenic role of GbE has been linked to competition with estrogens for ER-β binding, pointing GbE as a chemopreventive agent for breast cancer development, an important side effect of HRT (Oh and Chung, 2004, 2006).

In summary, ovariectomy increased body mass and adiposity and impaired the anorexigenic effect of serotonin. On the other hand, GbE reduced food intake and body adiposity, restored serotonin effectiveness, enhanced 5-HT levels of medial hypothalamus microdialysates and decreased 5-HTT hypothalamic levels. Our results suggest that GbE is a potential alternative therapy for the management of weight gain associated with menopause. More studies are necessary to understand the mechanisms involved in the beneficial role of *G. biloba* on the hypothalamic serotonin pathway.

## Author Contributions

RB, MT, and ER contributed to the study design and to the data acquisition, analysis and interpretation; drafted and revised the manuscript and provided final approval of the submitted version; responsible for all study aspects including its accuracy and integrity. IdA contributed to the data acquisition, analysis and interpretation; revised the manuscript and provided final approval of the submitted version; responsible for all study aspects including its accuracy and integrity. SC and LO contributed to the data analysis and interpretation; revised the manuscript and provided final approval of the submitted version; responsible for all study aspects including its accuracy and integrity.

## Conflict of Interest Statement

The authors declare that the research was conducted in the absence of any commercial or financial relationships that could be construed as a potential conflict of interest.

## Abbreviations

5-HIAA: 5-hydroxyindoleacetic acid
5-HT_1A_: serotonin receptor 1A
5-HT_1B_: serotonin receptor 1B
5-HT_2C_: serotonin receptor 2C
5-HTT: serotonin transporter
α-MSH: α-melanocyte-stimulating hormone
AgRP: agouti-related protein
AP: anteroposterior (stereotaxic coordinate)
CART: cocaine- and amphetamine-regulated transcript
DV: dorsoventral (stereotaxic coordinate)
ER: estrogen receptor
GbE: *Ginkgo biloba* (standardized) extract
GbE 761: *Ginkgo biloba* 761 standardized extract
HRT: hormone replacement therapy
kcal: kilocalorie
ML: mediolateral (stereotaxic coordinate)
NPY: neuropeptide Y
OVX: ovariectomized
POMC: pro-opiomelanocortin
Sham: false-ovariectomized
T°C: temperature in Celsius degrees
U: units
