# Supplementary figures and images for: Ginkgo biloba Extract (GbE) Stimulates the Hypothalamic Serotonergic System and Attenuates Obesity in Ovariectomized Rats
Source: Front Pharmacol. 2017 Sep 5;8:605. doi: 10.3389/fphar.2017.00605 (PMC5591947; doi:10.3389/fphar.2017.00605)

## 5-HT1A

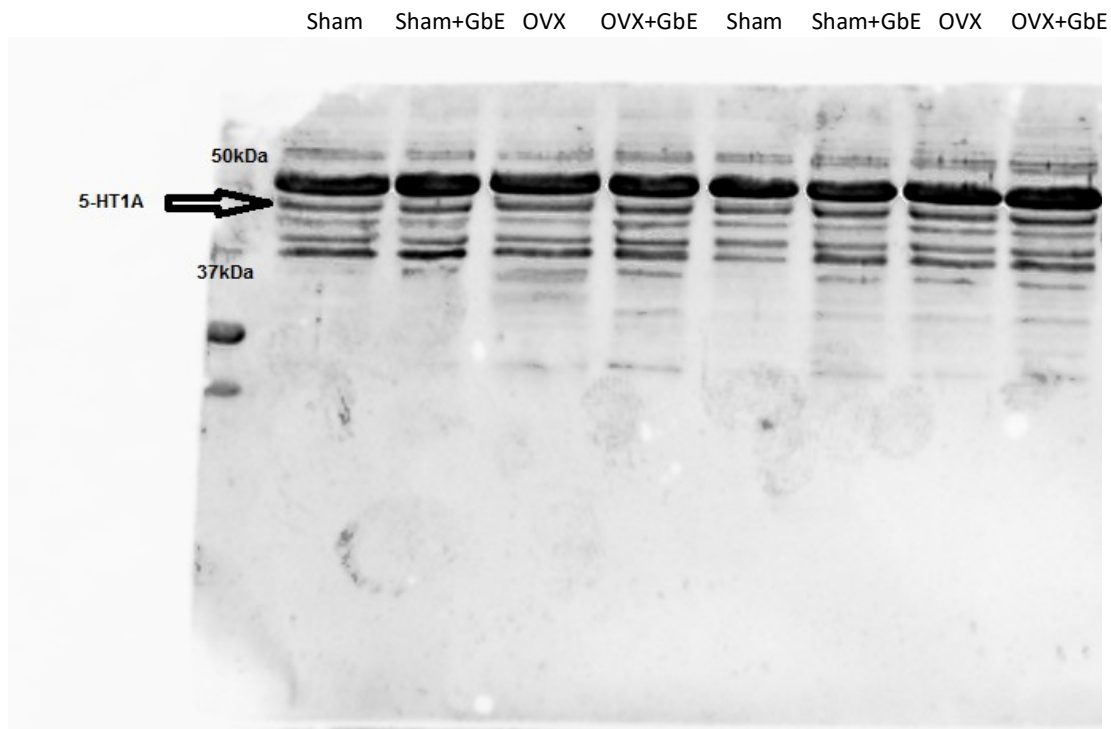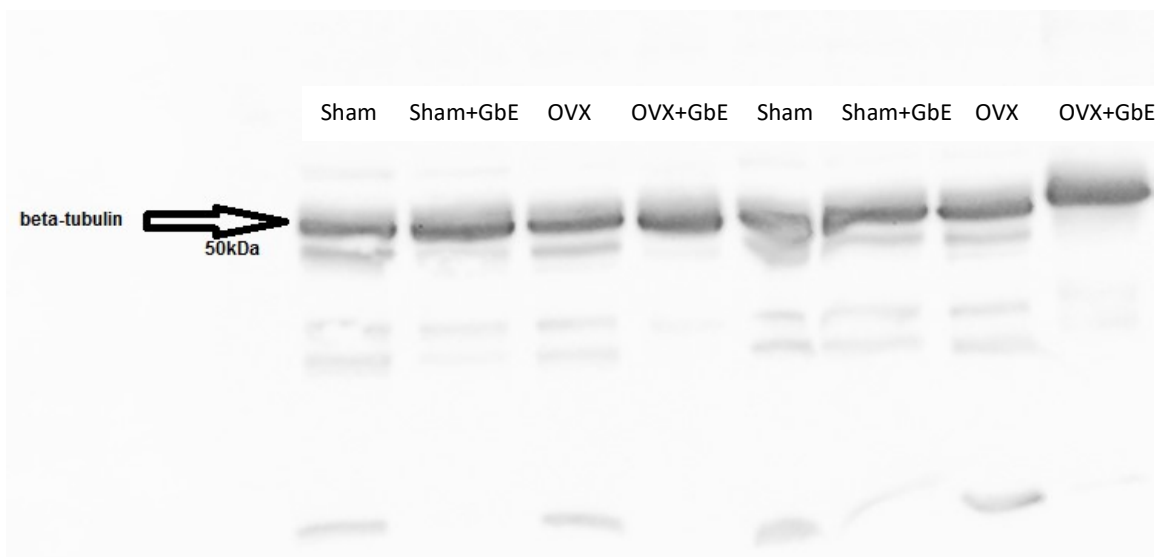

## 5-HT1B

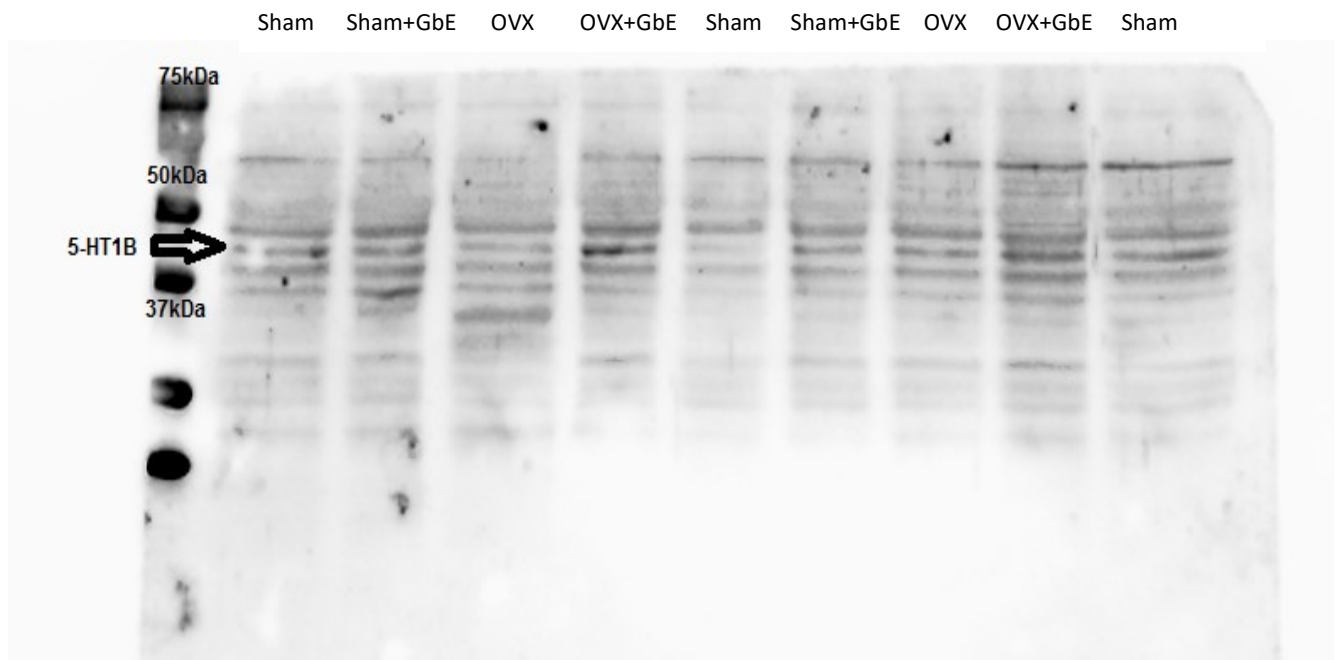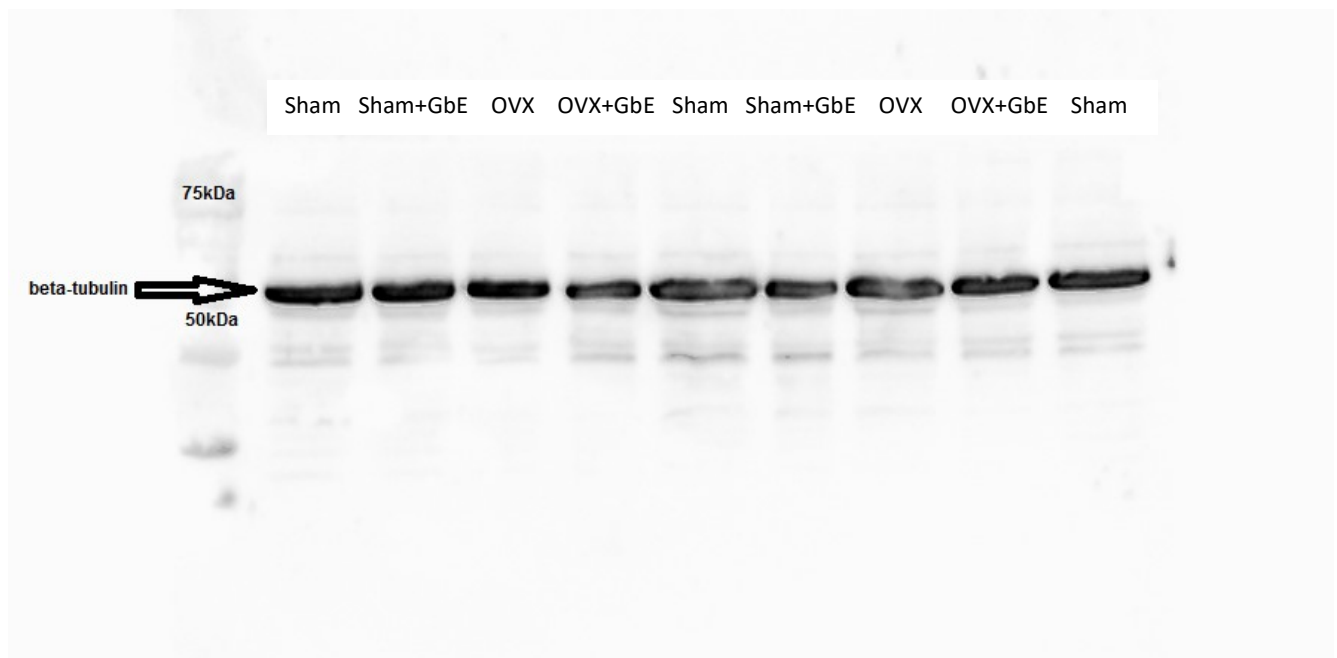

## 5-HT2C

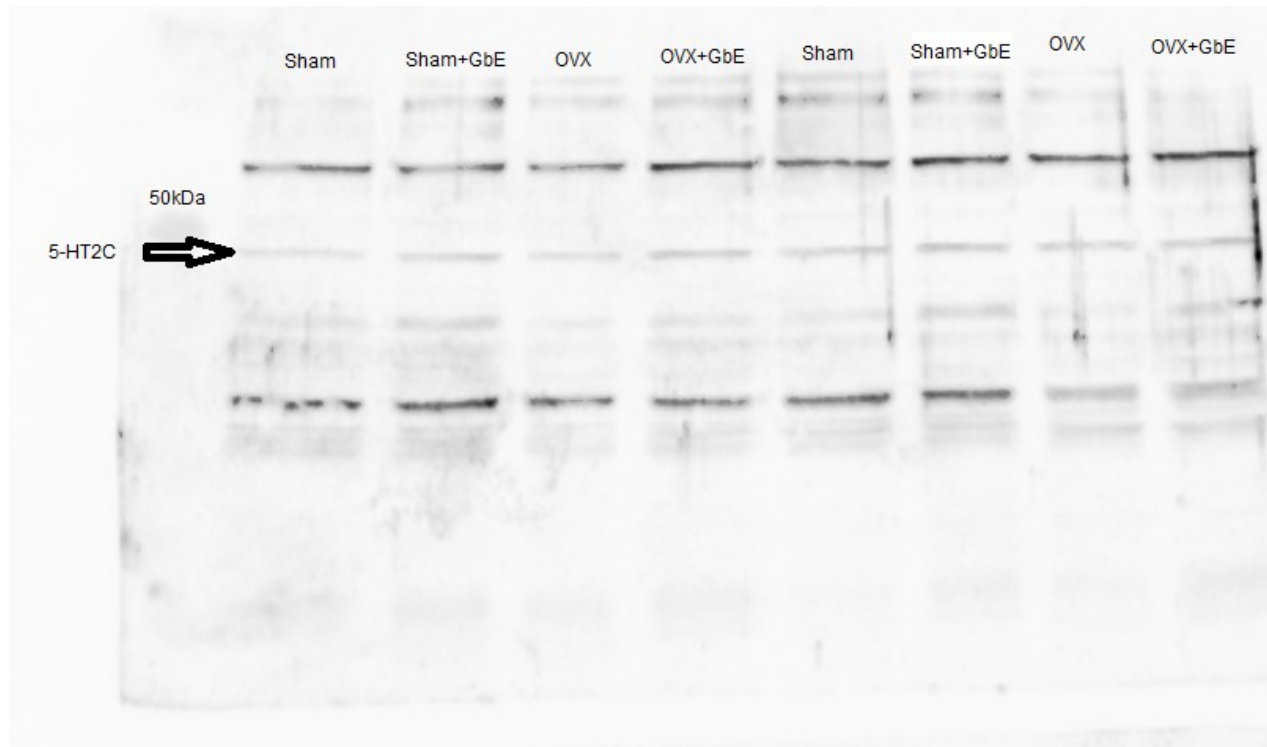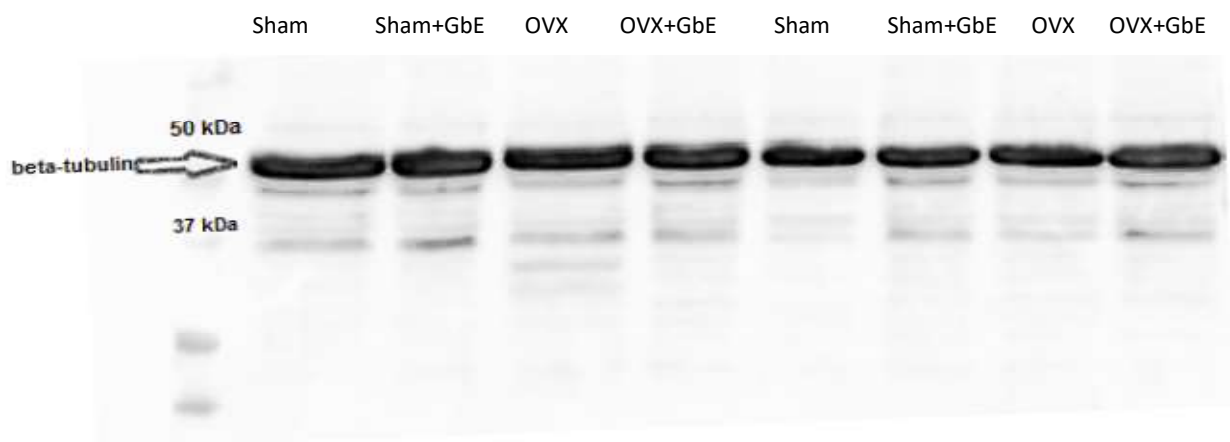

## 5-HTT

Sham Sham+GbE OVX OVX+GbE Sham Sham+GbE OVX OVX+GbE Sham

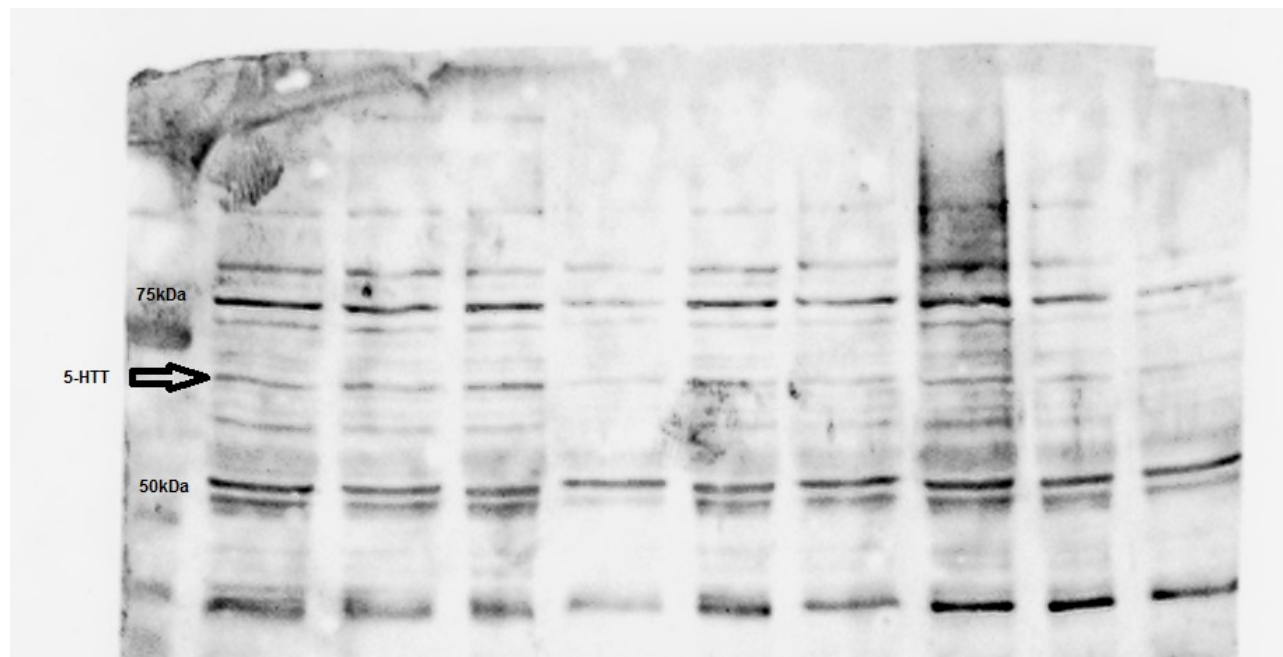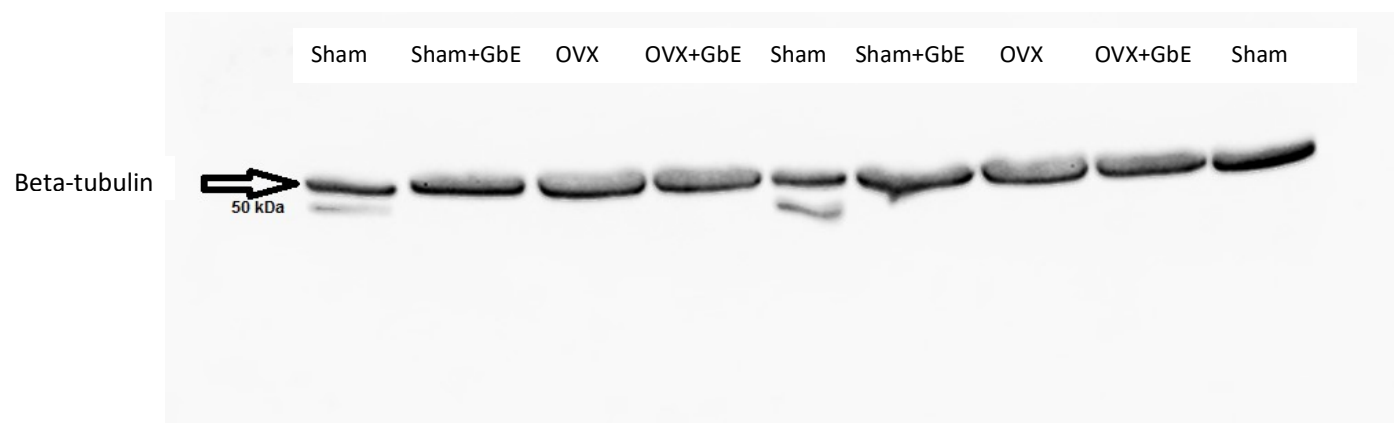

## POMC

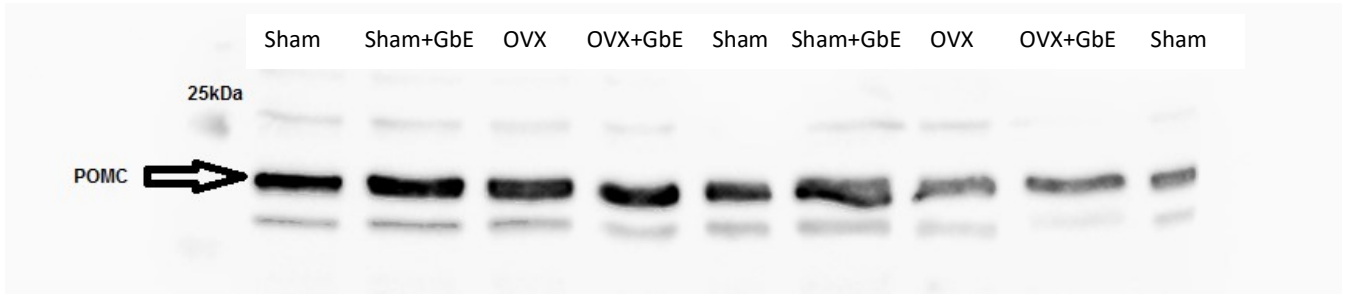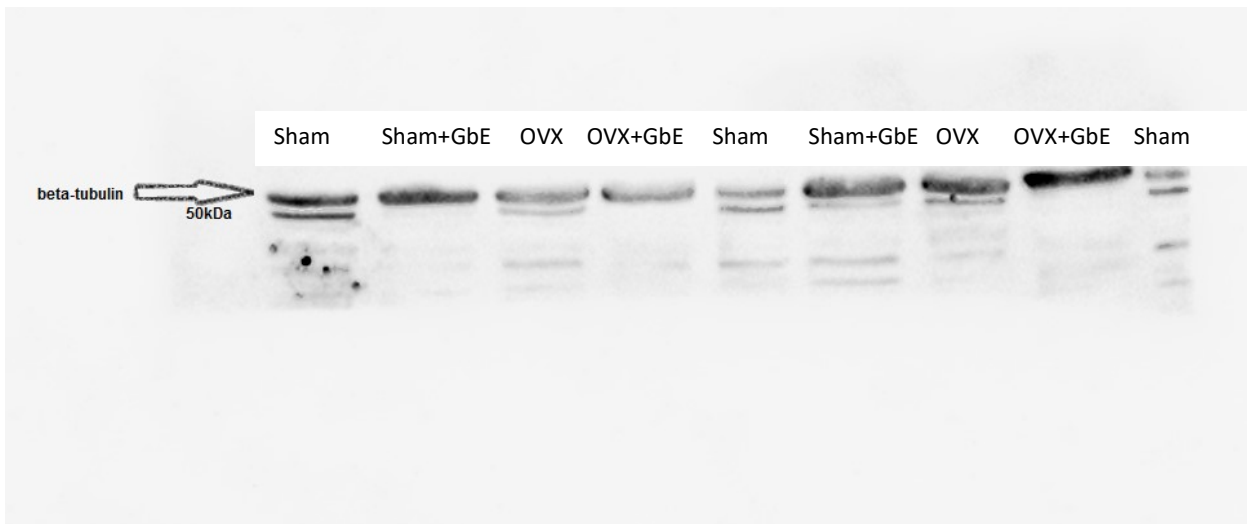

Supplement: Supplementary file 1 [file Data_Sheet_1.PDF]
