# Supplementary material for: Ginkgo biloba Extract (GbE) Stimulates the Hypothalamic Serotonergic System and Attenuates Obesity in Ovariectomized Rats
Source: Front Pharmacol. 2017 Sep 5;8:605. doi: 10.3389/fphar.2017.00605 (PMC5591947; doi:10.3389/fphar.2017.00605)
Supplement: Supplementary file 2 [file Data_Sheet_2.PDF]

Supplemental Material - Statistical Analysis

The body weight and food intake data measured weekly during the 8-week period after ovariectomy were analyzed by Student's *t*-test for independent samples between the groups and by the one-way ANOVA for repeated measures within each group.

Variable	Sham (n=48)				OVX (n=49)			
	Body weight (g)		Relative Food Intake (g/100g/24h)		Body weight (g)		Relative Food Intake (g/100g/24h)	
	Mean	SEM	Mean	SEM	Mean	SEM	Mean	SEM
**Week 0**	208.6	4.3	---	---	204.8	3.4	---	---
**Week 1**	220.1	3.9	8.5	0.3	224.8	4.1	9.6	0.1
**Week 2**	233.7	4.0	8.3	0.2	259.6	3.7	8.9	0.2
**Week 3**	242.3	3.9	7.6	0.3	283.5	3.3	8.0	0.2
**Week 4**	250.1	3.9	6.7	0.2	296.8	3.2	6.9	0.2
**Week 5**	256.9	4.0	7.4	0.4	307.1	2.9	6.8	0.1
**Week 6**	263.5	3.9	6.7	0.3	316.5	3.1	6.3	0.1
**Week 7**	266.7	4.0	6.7	0.2	323.9	3.3	6.1	0.1
**Week 8**	271.0	4.0	6.6	0.2	330.5	3.4	6.3	0.1

Legend: SEM (standard error mean)

Independent Samples Test

		Levene's Test for Equality of Variances		t-test for Equality of Means						
									95% Confidence Interval of the Difference	
		F	Sig.	t	df	Sig. (2-tailed)	Mean Difference	Std. Error Difference	Lower	Upper
body mass_week 0	Equal variances assumed	2,796	,098	,687	95	,494	3,81148	5,54814	-7,20296	14,82592
	Equal variances not assumed			,686	90,560	,495	3,81148	5,55977	-7,23304	14,85600
body mass_week 1	Equal variances assumed	,311	,578	-,828	95	,410	-4,69230	5,66737	-15,94345	6,55884
	Equal variances not assumed			-,828	94,923	,410	-4,69230	5,66446	-15,93779	6,55319
body mass_week 2	Equal variances assumed	,085	,771	-4,726	95	,000	-25,83889	5,46754	-36,69334	-14,98444
	Equal variances not assumed			-4,722	94,029	,000	-25,83889	5,47215	-36,70393	-14,97385
body mass_week 3	Equal variances assumed	,327	,569	-8,078	95	,000	-41,18882	5,09919	-51,31199	-31,06565
	Equal variances not assumed			-8,065	92,500	,000	-41,18882	5,10683	-51,33070	-31,04694
body mass_week 4	Equal variances assumed	1,363	,246	-9,214	95	,000	-46,69334	5,06746	-56,75352	-36,63316
	Equal variances not assumed			-9,196	90,918	,000	-46,69334	5,07758	-56,77945	-36,60723
body mass_week 5	Equal variances assumed	3,528	,063	-10,066	95	,000	-50,16733	4,98388	-60,06157	-40,27309
	Equal variances not assumed			-10,035	86,596	,000	-50,16733	4,99904	-60,10411	-40,23055
body mass_week 6	Equal variances assumed	,675	,413	-10,608	95	,000	-52,95036	4,99173	-62,86019	-43,04052
	Equal variances not assumed			-10,587	90,960	,000	-52,95036	5,00163	-62,88555	-43,01517
body mass_week 7	Equal variances assumed	1,006	,318	-11,052	95	,000	-57,27592	5,18236	-67,56421	-46,98763
	Equal variances not assumed			-11,030	91,033	,000	-57,27592	5,19254	-67,59021	-46,96163
body mass_week 8	Equal variances assumed	,429	,514	-11,250	95	,000	-59,43471	5,28314	-69,92307	-48,94635
	Equal variances not assumed			-11,233	92,364	,000	-59,43471	5,29131	-69,94316	-48,92627

Tests of Between-Subjects Effects

Measure: bodymass

Transformed Variable: Average

Source	Type III Sum of Squares	df	Mean Square	F	Sig.
Intercept	26119788,20	1	26119788,20	3928,550	,000
Error	312489,361	47	6648,710		

Pairwise Comparisons Sham group

Measure: bodymass

(I) factor1	(J) factor1	Mean Difference (I-J)	Std. Error	Sig.[b]	95% Confidence Interval for Difference[b] Lower Bound	Upper Bound
Week 0	1	-11,495*	1,120	,000	-15,301	-7,688
	2	-25,092*	1,487	,000	-30,145	-20,039
	3	-33,625*	1,282	,000	-37,982	-29,269
	4	-41,474*	1,333	,000	-46,005	-36,943
	5	-48,258*	1,292	,000	-52,648	-43,868
	6	-54,880*	1,659	,000	-60,518	-49,242
	7	-58,027*	1,739	,000	-63,939	-52,116
	8	-62,387*	1,830	,000	-68,606	-56,167
Week 1	0	11,495*	1,120	,000	7,688	15,301
	2	-13,597*	1,226	,000	-17,763	-9,432
	3	-22,131*	,951	,000	-25,364	-18,898
	4	-29,979*	1,084	,000	-33,663	-26,296
	5	-36,764*	1,237	,000	-40,969	-32,559
	6	-43,385*	1,465	,000	-48,363	-38,408
	7	-46,533*	1,499	,000	-51,628	-41,437
	8	-50,892*	1,636	,000	-56,453	-45,332
Week 2	0	25,092*	1,487	,000	20,039	30,145
	1	13,597*	1,226	,000	9,432	17,763
	3	-8,534*	1,147	,000	-12,433	-4,634
	4	-16,382*	1,191	,000	-20,431	-12,333
	5	-23,166*	1,291	,000	-27,553	-18,780
	6	-29,788*	1,512	,000	-34,927	-24,650
	7	-32,935*	1,550	,000	-38,203	-27,668
	8	-37,295*	1,726	,000	-43,162	-31,428
Week 3	0	33,625*	1,282	,000	29,269	37,982
	1	22,131*	,951	,000	18,898	25,364
	2	8,534*	1,147	,000	4,634	12,433
	4	-7,849*	,565	,000	-9,770	-5,927
	5	-14,633*	,862	,000	-17,561	-11,705
	6	-21,255*	1,161	,000	-25,199	-17,310
	7	-24,402*	,968	,000	-27,693	-21,111
	8	-28,761*	1,217	,000	-32,898	-24,624
Week 4	0	41,474*	1,333	,000	36,943	46,005
	1	29,979*	1,084	,000	26,296	33,663
	2	16,382*	1,191	,000	12,333	20,431
	3	7,849*	,565	,000	5,927	9,770
	5	-6,784*	,703	,000	-9,173	-4,395
	6	-13,406*	,975	,000	-16,721	-10,091
	7	-16,553*	,895	,000	-19,594	-13,512
	8	-20,913*	1,085	,000	-24,599	-17,226
Week 5	0	48,258*	1,292	,000	43,868	52,648
	1	36,764*	1,237	,000	32,559	40,969
	2	23,166*	1,291	,000	18,780	27,553
	3	14,633*	,862	,000	11,705	17,561
	4	6,784*	,703	,000	4,395	9,173
	6	-6,622*	,807	,000	-9,365	-3,878
	7	-9,769*	1,049	,000	-13,333	-6,205
	8	-14,128*	1,168	,000	-18,098	-10,158
Week 6	0	54,880*	1,659	,000	49,242	60,518
	1	43,385*	1,465	,000	38,408	48,363
	2	29,788*	1,512	,000	24,650	34,927
	3	21,255*	1,161	,000	17,310	25,199
	4	13,406*	,975	,000	10,091	16,721
	5	6,622*	,807	,000	3,878	9,365
	7	-3,147*	,872	,027	-6,110	-,184
	8	-7,507*	,822	,000	-10,299	-4,714
Week 7	0	58,027*	1,739	,000	52,116	63,939
	1	46,533*	1,499	,000	41,437	51,628
	2	32,935*	1,550	,000	27,668	38,203
	3	24,402*	,968	,000	21,111	27,693
	4	16,553*	,895	,000	13,512	19,594
	5	9,769*	1,049	,000	6,205	13,333
	6	3,147*	,872	,027	,184	6,110
	8	-4,359*	,618	,000	-6,458	-2,260
Week 8	0	62,387*	1,830	,000	56,167	68,606
	1	50,892*	1,636	,000	45,332	56,453
	2	37,295*	1,726	,000	31,428	43,162
	3	28,761*	1,217	,000	24,624	32,898
	4	20,913*	1,085	,000	17,226	24,599
	5	14,128*	1,168	,000	10,158	18,098
	6	7,507*	,822	,000	4,714	10,299
	7	4,359*	,618	,000	2,260	6,458

Tests of Between-Subjects Effects

Measure: bodymass

Transformed Variable: Average

Source	Type III Sum of Squares	df	Mean Square	F	Sig.
Intercept	35331232,24	1	35331232,24	7808,627	,000
Error	217182,745	48	4524,641		

Pairwise Comparisons - OVX group

Measure: bodymass

(I) factor1bodymass	(J) factor1bodymass	Mean Difference (I-J)	Std. Error	Sig.[b]	95% Confidence[b]... Lower Bound
Week 0	1	-19,998*	1,323	,000	-24,488
	2	-54,742*	1,434	,000	-59,611
	3	-78,626*	1,530	,000	-83,817
	4	-91,979*	1,669	,000	-97,643
	5	-102,237*	2,067	,000	-109,251
	6	-111,642*	2,299	,000	-119,446
	7	-119,072*	2,407	,000	-127,242
	8	-125,656*	2,605	,000	-134,499
Week 1	0	19,998*	1,323	,000	15,509
	2	-34,744*	1,160	,000	-38,680
	3	-58,627*	1,635	,000	-64,176
	4	-71,980*	1,988	,000	-78,728
	5	-82,239*	2,504	,000	-90,736
	6	-91,643*	2,585	,000	-100,419
	7	-99,074*	2,765	,000	-108,461
	8	-105,658*	2,894	,000	-115,481
Week 2	0	54,742*	1,434	,000	49,873
	1	34,744*	1,160	,000	30,808
	3	-23,883	1,506	,000	-28,994
	4	-37,237*	1,712	,000	-43,047
	5	-47,495*	2,014	,000	-54,329
	6	-56,900*	2,105	,000	-64,045
	7	-64,330*	2,351	,000	-72,309
	8	-70,914*	2,474	,000	-79,312
Week 3	0	78,626*	1,530	,000	73,434
	1	58,627*	1,635	,000	53,078
	2	23,883*	1,506	,000	18,773
	4	-13,353*	,874	,000	-16,321
	5	-23,611*	1,501	,000	-28,706
	6	-33,016*	1,508	,000	-38,134
	7	-40,447*	1,845	,000	-46,708
	8	-47,031*	1,926	,000	-53,567
Week 4	0	91,979*	1,669	,000	86,315
	1	71,980*	1,988	,000	65,233
	2	37,237*	1,712	,000	31,426
	3	13,353*	,874	,000	10,385
	5	-10,258*	,909	,000	-13,345
	6	-19,663*	1,026	,000	-23,145
	7	-27,094*	1,258	,000	-31,364
	8	-33,678*	1,414	,000	-38,475
Week 5	0	102,237*	2,067	,000	95,223
	1	82,239*	2,504	,000	73,741
	2	47,495*	2,014	,000	40,660
	3	23,611*	1,501	,000	18,517
	4	10,258*	,909	,000	7,172
	6	-9,405*	,661	,000	-11,649
	7	-16,835*	,876	,000	-19,810
	8	-23,419*	1,011	,000	-26,851
Week 6	0	111,642*	2,299	,000	103,838
	1	91,643*	2,585	,000	82,868
	2	56,900*	2,105	,000	49,754
	3	33,016*	1,508	,000	27,898
	4	19,663*	1,026	,000	16,181
	5	9,405*	,661	,000	7,160
	7	-7,431*	,798	,000	-10,139
	8	-14,014*	,883	,000	-17,011
Week 7	0	119,072*	2,407	,000	110,903
	1	99,074*	2,765	,000	89,688
	2	64,330*	2,351	,000	56,351
	3	40,447*	1,845	,000	34,185
	4	27,094*	1,258	,000	22,824
	5	16,835*	,876	,000	13,861
	6	7,431*	,798	,000	4,722
	8	-6,584*	,660	,000	-8,825
9	0	125,656*	2,605	,000	116,814
	1	105,658*	2,894	,000	95,835
	2	70,914*	2,474	,000	62,516
	3	47,031*	1,926	,000	40,494
	4	33,678*	1,414	,000	28,880
	5	23,419*	1,011	,000	19,987
	6	14,014*	,883	,000	11,018
	7	6,584*	,660	,000	4,343

Independent Samples Test

		Levene's Test for Equality of Variances		t-test for Equality of Means					95% Confidence Interval of the Difference	
		F	Sig.	t	df	Sig. (2-tailed)	Mean Difference	Std. Error Difference	Lower	Upper
relative food intake_week 1	Equal variances assumed	10,413	,004	-3,064	22	,006	-1,08441	,35388	-1,81832	-,35049
	Equal variances not assumed			-3,240	17,570	,005	-1,08441	,33464	-1,78870	-,38011
relative food intake_week 2	Equal variances assumed	,519	,479	-2,461	23	,022	-,72782	,29578	-1,33969	-,11596
	Equal variances not assumed			-2,485	22,456	,021	-,72782	,29287	-1,33448	-,12117
relative food intake_week 3	Equal variances assumed	6,487	,018	-1,227	22	,233	-,45538	,37107	-1,22495	,31418
	Equal variances not assumed			-1,277	20,238	,216	-,45538	,35672	-1,19892	,28815
relative food intake_week 4	Equal variances assumed	,140	,712	-,564	22	,578	-,17083	,30292	-,79905	,45738
	Equal variances not assumed			-,564	20,039	,579	-,17083	,30292	-,80263	,46096
relative food intake_week 5	Equal variances assumed	10,931	,003	1,292	23	,209	,55462	,42925	-,33336	1,44259
	Equal variances not assumed			1,338	14,241	,202	,55462	,41449	-,33297	1,44221
relative food intake_week 6	Equal variances assumed	36,449	,000	1,522	24	,141	,46923	,30827	-,16701	1,10547
	Equal variances not assumed			1,522	14,872	,149	,46923	,30827	-,18833	1,12679
relative food intake_week 7	Equal variances assumed	,058	,812	2,824	24	,009	,59905	,21213	,16123	1,03686
	Equal variances not assumed			2,797	22,254	,010	,59905	,21419	,15513	1,04296
relative food intake_week 8	Equal variances assumed	5,338	,029	1,192	25	,244	,28363	,23791	-,20635	,77360
	Equal variances not assumed			1,173	20,270	,254	,28363	,24172	-,22016	,78741

Tests of Between-Subjects Effects

Measure: foodintake

Transformed Variable: Average

Source	Type III Sum of Squares	df	Mean Square	F	Sig.
Intercept	4582,391	1	4582,391	1577,102	,000
Error	29,056	10	2,906		

Pairwise Comparisons - Sham group

Measure foodintake

(I) factor1foodintake	(J) factor1foodintake	Mean Difference (I-J)	Std. Error	Sig.[b]	95% Confidence Interval for Difference[b] Lower Bound	Upper Bound
1	2	,124	,253	1,000	-,944	1,191
	3	,825	,283	,428	-,366	2,017
	4	1,605*	,321	,015	,251	2,960
	5	1,137	,593	1,000	-1,363	3,638
	6	1,724*	,273	,002	,572	2,876
	7	1,566*	,231	,001	,594	2,539
	8	1,864*	,177	,000	1,119	2,608
2	1	-,124	,253	1,000	-1,191	,944
	3	,702	,201	,164	-,146	1,550
	4	1,482*	,329	,032	,094	2,869
	5	1,014	,564	1,000	-1,364	3,392
	6	1,600*	,153	,000	,955	2,245
	7	1,443*	,197	,001	,611	2,274
	8	1,740*	,193	,000	,925	2,555
3	1	-,825	,283	,428	-2,017	,366
	2	-,702	,201	,164	-1,550	,146
	4	,780	,354	1,000	-,713	2,273
	5	,312	,614	1,000	-2,278	2,902
	6	,898*	,176	,013	,156	1,641
	7	,741	,257	,453	-,341	1,822
	8	1,038	,263	,076	-,069	2,146
4	1	-1,605*	,321	,015	-2,960	-,251
	2	-1,482*	,329	,032	-2,869	-,094
	3	-,780	,354	1,000	-2,273	,713
	5	-,468	,473	1,000	-2,463	1,526
	6	,118	,333	1,000	-1,286	1,523
	7	-,039	,245	1,000	-1,073	,994
	8	,258	,350	1,000	-1,216	1,733
5	1	-1,137	,593	1,000	-3,638	1,363
	2	-1,014	,564	1,000	-3,392	1,364
	3	-,312	,614	1,000	-2,902	2,278
	4	,468	,473	1,000	-1,526	2,463
	6	,586	,610	1,000	-1,985	3,157
	7	,429	,457	1,000	-1,497	2,355
	8	,726	,565	1,000	-1,656	3,109
6	1	-1,724*	,273	,002	-2,876	-,572
	2	-1,600*	,153	,000	-2,245	-,955
	3	-,898*	,176	,013	-1,641	-,156
	4	-,118	,333	1,000	-1,523	1,286
	5	-,586	,610	1,000	-3,157	1,985
	7	-,157	,270	1,000	-1,295	,980
	8	,140	,276	1,000	-1,022	1,302
7	1	-1,566*	,231	,001	-2,539	-,594
	2	-1,443*	,197	,001	-2,274	-,611
	3	-,741	,257	,453	-1,822	,341
	4	,039	,245	1,000	-,994	1,073
	5	-,429	,457	1,000	-2,355	1,497
	6	,157	,270	1,000	-,980	1,295
	8	,297	,181	1,000	-,465	1,059
8	1	-1,864*	,177	,000	-2,608	-1,119
	2	-1,740*	,193	,000	-2,555	-,925
	3	-1,038	,263	,076	-2,146	,069
	4	-,258	,350	1,000	-1,733	1,216
	5	-,726	,565	1,000	-3,109	1,656
	6	-,140	,276	1,000	-1,302	1,022
	7	-,297	,181	1,000	-1,059	,465

Based on estimated marginal means

*. The mean difference is significant at the ,05 level.

b. Adjustment for multiple comparisons: Bonferroni.

Tests of Between-Subjects Effects

Measure: foodintake

Transformed Variable: Average

Source	Type III Sum of Squares	df	Mean Square	F	Sig.
Intercept	3831,626	1	3831,626	4712,820	,000
Error	6,504	8	,813		

Pairwise Comparisons - OVX group

Measure foodintake

(I) factor1foodintake	(J) factor1foodintake	Mean Difference (I-J)	Std. Error	Sig.[b]	95% Confidence Interval for Difference[b] Lower Bound	Upper Bound
1	2	,883*	,189	,044	,018	1,749
	3	1,754*	,219	,001	,751	2,758
	4	2,770*	,209	,000	1,812	3,728
	5	2,997*	,222	,000	1,977	4,016
	6	3,518*	,101	,000	3,054	3,982
	7	3,688*	,222	,000	2,671	4,705
	8	3,568*	,222	,000	2,551	4,584
2	1	-,883*	,189	,044	-1,749	-,018
	3	,871	,266	,317	-,350	2,092
	4	1,887*	,247	,002	,752	3,021
	5	2,113*	,225	,000	1,082	3,144
	6	2,634*	,179	,000	1,813	3,456
	7	2,804*	,157	,000	2,086	3,523
	8	2,684*	,244	,000	1,566	3,803
3	1	-1,754*	,219	,001	-2,758	-,751
	2	-,871	,266	,317	-2,092	,350
	4	1,016*	,076	,000	,668	1,363
	5	1,242*	,234	,020	,169	2,315
	6	1,763*	,186	,000	,908	2,619
	7	1,933*	,286	,004	,622	3,244
	8	1,813*	,240	,002	,711	2,915
4	1	-2,770*	,209	,000	-3,728	-1,812
	2	-1,887*	,247	,002	-3,021	-,752
	3	-1,016*	,076	,000	-1,363	-,668
	5	,227	,255	1,000	-,944	1,398
	6	,748	,200	,162	-,172	1,667
	7	,918	,273	,279	-,336	2,172
	8	,798	,256	,400	-,376	1,971
5	1	-2,997*	,222	,000	-4,016	-1,977
	2	-2,113*	,225	,000	-3,144	-1,082
	3	-1,242*	,234	,020	-2,315	-,169
	4	-,227	,255	1,000	-1,398	,944
	6	,521	,195	,793	-,374	1,416
	7	,691	,177	,125	-,120	1,502
	8	,571	,162	,219	-,173	1,315
6	1	-3,518*	,101	,000	-3,982	-3,054
	2	-2,634*	,179	,000	-3,456	-1,813
	3	-1,763*	,186	,000	-2,619	-,908
	4	-,748	,200	,162	-1,667	,172
	5	-,521	,195	,793	-1,416	,374
	7	,170	,228	1,000	-,878	1,218
	8	,050	,223	1,000	-,971	1,071
7	1	-3,688*	,222	,000	-4,705	-2,671
	2	-2,804*	,157	,000	-3,523	-2,086
	3	-1,933*	,286	,004	-3,244	-,622
	4	-,918	,273	,279	-2,172	,336
	5	-,691	,177	,125	-1,502	,120
	6	-,170	,228	1,000	-1,218	,878
	8	-,120	,160	1,000	-,852	,612
8	1	-3,568*	,222	,000	-4,584	-2,551
	2	-2,684*	,244	,000	-3,803	-1,566
	3	-1,813*	,240	,002	-2,915	-,711
	4	-,798	,256	,400	-1,971	,376
	5	-,571	,162	,219	-1,315	,173
	6	-,050	,223	1,000	-1,071	,971
	7	,120	,160	1,000	-,612	,852

Based on estimated marginal means

*. The mean difference is significant at the ,05 level.

b. Adjustment for multiple comparisons: Bonferroni.

The body weight gain (%) and food efficiency data during the 8-week period after ovariectomy were analyzed by Student's *t*-test for independent samples.

Sham (n=48)				OVX (n=49)			
**Body weight gain (%)**		**Food Efficiency ($g_{BW\ gain}/g_{food\ consumption}$)**		**Body weight gain (%)**		**Food Efficiency ($g_{BW\ gain}/g_{food\ consumption}$)**	
Mean	SEM	Mean	SEM	Mean	SEM	Mean	SEM
23.4	0.9	0.15	0.01	48.6	2.2	0.39	0.05

Independent Samples Test

		t-test for Equality of Means		
		Sig. (2-tailed)	Mean Difference	Std. Error Difference
body weight gain (%)	Equal variances assumed	,000	-25,14791	2,37251
	Equal variances not assumed	,000	-25,14791	2,35503

Independent Samples Test

		t-test for Equality of Means				
		t	df	Sig. (2-tailed)	Mean Difference	Std. Error Difference
food efficiency	Equal variances assumed	-5,664	18	,000	-30,49071	5,38342
	Equal variances not assumed	-5,208	9,538	,000	-30,49071	5,85501

Sham (n=13)				Sham+GbE (n=12)				OVX (n=14)				OVX+GbE (n=12)			
**Body weight gain (%)**		**Cumulative food intake (g/100g/24h)**		**Body weight gain (%)**		**Cumulative food intake (g/100g/24h)**		**Body weight gain (%)**		**Cumulative food intake (g/100g/24h)**		**Body weight gain (%)**		**Cumulative food intake (g/100g/24h)**	
Mean	SEM	Mean	SEM	Mean	SEM	Mean	SEM	Mean	SEM	Mean	SEM	Mean	SEM	Mean	SEM
1.5	0.5	6.1	0.2	0.9	0.3	6.0	0.2	1.6	0.4	5.6	0.2	1.1	0.4	5.2	0.1

Univariate Analysis of Variance

[DataSet0]

Between-Subjects Factors

		N
Sham or OVX	1	25
	2	26
Vehicle or GbE	1	27
	2	24

Tests of Between-Subjects Effects

Dependent Variable: body weight gain (%)

Source	Type III Sum of Squares	df	Mean Square	F	Sig.
Corrected Model	4,595[a]	3	1,532	,624	,603
Intercept	83,870	1	83,870	34,162	,000
OVX	,361	1	,361	,147	,703
GbE	4,066	1	4,066	1,656	,204
OVX * GbE	,179	1	,179	,073	,788
Error	115,388	47	2,455		
Total	206,459	51			
Corrected Total	119,982	50			

a. R Squared = ,038 (Adjusted R Squared = -,023)

Univariate Analysis of Variance

[DataSet0]

Between-Subjects Factors

		N
Sham or OVX	1	11
	2	11
Vehicle or GbE	1	10
	2	12

Tests of Between-Subjects Effects

Dependent Variable: cumulative food intake

Source	Type III Sum of Squares	df	Mean Square	F	Sig.
Corrected Model	3,095[a]	3	1,032	4,993	,011
Intercept	711,778	1	711,778	3445,036	,000
OVX	2,542	1	2,542	12,303	,003
GbE	,280	1	,280	1,356	,259
OVX * GbE	,141	1	,141	,681	,420
Error	3,719	18	,207		
Total	721,936	22			
Corrected Total	6,814	21			

a. R Squared = ,454 (Adjusted R Squared = ,363)

Multiple Comparisons

Dependent Variable: cumulative food intake

Tukey HSD

(I) experimental groups	(J) experimental groups	Mean Difference (I-J)	Std. Error	Sig.	95% Confidence Interval Lower Bound	95% Confidence Interval Upper Bound
1	2	,0660	,27524	,995	-,7119	,8439
	3	,5220	,28748	,298	-,2905	1,3345
	4	,9093*	,27524	,019	,1314	1,6872
2	1	-,0660	,27524	,995	-,8439	,7119
	3	,4560	,27524	,374	-,3219	1,2339
	4	,8433*	,26243	,023	,1016	1,5850
3	1	-,5220	,28748	,298	-1,3345	,2905
	2	-,4560	,27524	,374	-1,2339	,3219
	4	,3873	,27524	,511	-,3906	1,1652
4	1	-,9093*	,27524	,019	-1,6872	-,1314
	2	-,8433*	,26243	,023	-1,5850	-,1016
	3	-,3873	,27524	,511	-1,1652	,3906

Body weight gain (%), body adiposity (g/100g), cumulative food intake and uterus mass data during the phytotherapy treatment were analyzed by two-way ANOVA with OVX and GbE as main factors.

Group	Uterus mass (g)		Retroperitoneal adipose tissue depot (g/100g)		Mesenteric adipose tissue depot (g/100g)		Sum of visceral adipose tissues depots (g/100g)	
	Mean	SEM	Mean	SEM	Mean	SEM	Mean	SEM
**Sham (n=21-24)**	0.52	0.018	0.69	0.051	0.47	0.041	1.16	0.085
**Sham+GbE (n=21-23)**	0.50	0.023	0.61	0.057	0.36	0.032	0.96	0.077
**OVX (n=18-25)**	0.14	0.006	1.24	0.072	0.77	0.039	2.01	0.101
**OVX+GbE (n=18-23)**	0.15	0.005	0.96	0.085	0.69	0.063	1.60	0.140

Univariate Analysis of Variance

[DataSet0]

Between-Subjects Factors

		N
Sham or OVX	1	47
	2	48
Vehicle or GbE	1	49
	2	46

Tests of Between-Subjects Effects

Dependent Variable: Uterus mass

Source	Type III Sum of Squares	df	Mean Square	F	Sig.
Corrected Model	3,215[a]	3	1,072	195,730	,000
Intercept	10,180	1	10,180	1859,078	,000
OVX	3,201	1	3,201	584,461	,000
GbE	,001	1	,001	,138	,711
OVX * GbE	,004	1	,004	,789	,377
Error	,498	91	,005		
Total	13,788	95			
Corrected Total	3,714	94			

a. R Squared = ,866 (Adjusted R Squared = ,861)

Post Hoc Tests

experimental group

Multiple Comparisons

Dependent Variable: Uterus mass
Tukey HSD

(I) experimental group	(J) experimental group	Mean Difference (I-J)	Std. Error	Sig.	95% Confidence Interval Lower Bound	95% Confidence Interval Upper Bound
1	2	,0192	,02159	,812	-,0374	,0757
	3	,3808*	,02115	,000	,3255	,4362
	4	,3730*	,02159	,000	,3165	,4295
2	1	-,0192	,02159	,812	-,0757	,0374
	3	,3617*	,02138	,000	,3057	,4176
	4	,3538*	,02182	,000	,2967	,4109
3	1	-,3808*	,02115	,000	-,4362	-,3255
	2	-,3617*	,02138	,000	-,4176	-,3057
	4	-,0078	,02138	,983	-,0638	,0481
4	1	-,3730*	,02159	,000	-,4295	-,3165
	2	-,3538*	,02182	,000	-,4109	-,2967
	3	,0078	,02138	,983	-,0481	,0638

Based on observed means.
The error term is Mean Square(Error) = ,005.
*. The mean difference is significant at the 0,05 level.

Univariate Analysis of Variance

[DataSet0]

Between-Subjects Factors

		N
Sham or OVX	1	43
	2	36
Vehicle or GbE	1	39
	2	40

Tests of Between-Subjects Effects

Dependent Variable: Retroperitoneal fat pad

Source	Type III Sum of Squares	df	Mean Square	F	Sig.
Corrected Model	4,702[a]	3	1,567	18,311	,000
Intercept	60,442	1	60,442	706,084	,000
OVX	3,908	1	3,908	45,659	,000
GbE	,640	1	,640	7,472	,008
OVX * GbE	,193	1	,193	2,250	,138
Error	6,420	75	,086		
Total	69,273	79			
Corrected Total	11,122	78			

a. R Squared = ,423 (Adjusted R Squared = ,400)

Post Hoc Tests

experimental group

Multiple Comparisons

Dependent Variable: Retroperitoneal fat pad
Tukey HSD

(I) experimental group	(J) experimental group	Mean Difference (I-J)	Std. Error	Sig.	95% Confidence Interval Lower Bound	95% Confidence Interval Upper Bound
1	2	,0815	,08926	,798	-,1530	,3161
	3	-,5458*	,09398	,000	-,7928	-,2989
	4	-,2660*	,09398	,030	-,5129	-,0190
2	1	-,0815	,08926	,798	-,3161	,1530
	3	-,6274*	,09299	,000	-,8717	-,3830
	4	-,3475*	,09299	,002	-,5918	-,1032
3	1	,5458*	,09398	,000	,2989	,7928
	2	,6274*	,09299	,000	,3830	,8717
	4	,2799*	,09753	,027	,0236	,5361
4	1	,2660*	,09398	,030	,0190	,5129
	2	,3475*	,09299	,002	,1032	,5918
	3	-,2799*	,09753	,027	-,5361	-,0236

Based on observed means.
The error term is Mean Square(Error) = ,086.
*. The mean difference is significant at the 0,05 level.

Legend: 1 (Sham group); 2 (Sham+GbE group); 3 (OVX group); 4 (OVX+GbE group)

Univariate Analysis of Variance

[DataSet0]

Between-Subjects Factors

		N
Sham or OVX	1	42
	2	37
Vehicle or GbE	1	40
	2	39

Tests of Between-Subjects Effects

Dependent Variable: Mesenteric fat pad

Source	Type III Sum of Squares	df	Mean Square	F	Sig.
Corrected Model	2,108[a]	3	,703	18,139	,000
Intercept	25,572	1	25,572	660,201	,000
OVX	1,915	1	1,915	49,440	,000
GbE	,171	1	,171	4,417	,039
OVX * GbE	,004	1	,004	,097	,756
Error	2,905	75	,039		
Total	29,860	79			
Corrected Total	5,013	78			

a. R Squared = ,420 (Adjusted R Squared = ,397)

Post Hoc Tests

experimental group

Multiple Comparisons

Dependent Variable: Mesenteric fat pad
Tukey HSD

(I) experimental group	(J) experimental group	Mean Difference (I-J)	Std. Error	Sig.	95% Confidence Interval	
					Lower Bound	Upper Bound
1	2	,1071	,06074	,299	-,0525	,2667
	3	-,2982*	,06231	,000	-,4620	-,1345
	4	-,2188*	,06322	,005	-,3849	-,0527
2	1	-,1071	,06074	,299	-,2667	,0525
	3	-,4053*	,06231	,000	-,5691	-,2416
	4	-,3259*	,06322	,000	-,4920	-,1598
3	1	,2982*	,06231	,000	,1345	,4620
	2	,4053*	,06231	,000	,2416	,5691
	4	,0794	,06473	,612	-,0907	,2495
4	1	,2188*	,06322	,005	,0527	,3849
	2	,3259*	,06322	,000	,1598	,4920
	3	-,0794	,06473	,612	-,2495	,0907

Based on observed means.
The error term is Mean Square(Error) = ,039.

*. The mean difference is significant at the 0,05 level.

Univariate Analysis of Variance

[DataSet0]

Between-Subjects Factors

		N
Sham or OVX	1	43
	2	36
Vehicle or GbE	1	39
	2	40

Tests of Between-Subjects Effects

Dependent Variable: Sum of RET and MET fat pads

Source	Type III Sum of Squares	df	Mean Square	F	Sig.
Corrected Model	12,961[a]	3	4,320	21,757	,000
Intercept	161,151	1	161,151	811,577	,000
OVX	10,912	1	10,912	54,956	,000
GbE	1,862	1	1,862	9,377	,003
OVX * GbE	,209	1	,209	1,051	,308
Error	14,892	75	,199		
Total	182,627	79			
Corrected Total	27,853	78			

a. R Squared = ,465 (Adjusted R Squared = ,444)

Post Hoc Tests

experimental group

Multiple Comparisons

Dependent Variable: Sum of RET and MET fat pads
Tukey HSD

(I) experimental group	(J) experimental group	Mean Difference (I-J)	Std. Error	Sig.	95% Confidence Interval	
					Lower Bound	Upper Bound
1	2	,2051	,13595	,438	-,1522	,5623
	3	-,8496*	,14313	,000	-1,2257	-,4735
	4	-,4381*	,14313	,016	-,8141	-,0620
2	1	-,2051	,13595	,438	-,5623	,1522
	3	-1,0546*	,14162	,000	-1,4268	-,6825
	4	-,6431*	,14162	,000	-1,0152	-,2710
3	1	,8496*	,14313	,000	,4735	1,2257
	2	1,0546*	,14162	,000	,6825	1,4268
	4	,4115*	,14854	,035	,0212	,8018
4	1	,4381*	,14313	,016	,0620	,8141
	2	,6431*	,14162	,000	,2710	1,0152
	3	-,4115*	,14854	,035	-,8018	-,0212

Based on observed means.
The error term is Mean Square(Error) = ,199.

*. The mean difference is significant at the 0,05 level.

Legend: 1 (Sham group); 2 (Sham+GbE group); 3 (OVX group); 4 (OVX+GbE group)

Acute food intake after an i.c.v. injection of serotonin was evaluated by the Paired Student's *t*-test.

Time window	Sham group (n=4)		Sham+GbE group (n=4)		OVX group (n=3)		OVX+GbE group (n=5)	
	Vehicle	5-HT	Vehicle	5-HT	Vehicle	5-HT	Vehicle	5-HT
**0-12 hours**	5.66±0.86	3.26±0.39	5.61±0.34	4.81±0.29	5.27±0.10	5.13±0.29	3.33±0.40	2.03±0.72
**0-24 hours**	8.53±0.38	6.37±0.39	8.58±0.44	7.28±0.04	7.75±0.70	7.43±0.71	5.36±0.41	3.11±0.90

Paired Samples Test

		Paired ... 95% Confidence ... Upper	t	df	Sig. (2-tailed)
Pair 1	Food intake_vehicle_12h_Sham group - Food intake_serotonin_12h_Sham group	3,98657	4,302	3	,017
Pair 2	Food intake_vehicle_24h_Sham group - Food intake_serotonin_24h_Sham group	2,58867	15,598	3	,001
Pair 3	Food intake_vehicle_12h_Sham+GbE group - Food intake_serotonin_12h_Sham+GbE group	1,39984	4,214	3	,024
Pair 4	Food intake_vehicle_24h_Sham+GbE group - Food intake_serotonin_24h_Sham+GbE group	2,66039	3,495	3	,040
Pair 5	Food intake_vehicle_12h_OVX group - Food intake_serotonin_12h_OVX group	1,28675	,525	2	,652
Pair 6	Food intake_vehicle_24h_OVX group - Food intake_serotonin_24h_OVX group	1,46513	1,235	2	,342
Pair 7	Food intake_vehicle_12h_OVX+GbE group - Food intake_serotonin_12h_OVX+GbE group	2,57184	2,338	4	,047
Pair 8	Food intake_vehicle_24h_OVX+GbE group - Food intake_serotonin_24h_OVX+GbE group	5,18020	3,079	4	,037

Basal extracellular serotonin levels by time among the different groups were analyzed by two-way ANOVA, with OVX and GbE as the main factors.

Time (minutes)	Sham group (n=6-7)		Sham+GbE group (n=5-6)		OVX group (n=5-10)		OVX+GbE group (n=7-10)	
	Mean	SEM	Mean	SEM	Mean	SEM	Mean	SEM
**-40**	105.12	3.56	106.92	7.80	100.98	2.66	98.72	3.69
**-20**	100.67	4.08	101.66	8.39	98.67	3.91	99.45	3.72
**0**	93.25	6.40	91.42	5.95	100.56	4.16	96.60	4.57
**20**	99.11	8.83	125.45	20.92	75.18	7.36	123.52	11.57
**40**	94.20	9.14	88.46	17.22	95.72	15.56	141.10	15.06
**60**	97.89	6.09	108.47	18.68	98.16	20.94	159.72	24.21
**80**	75.35	7.48	95.47	14.11	85.74	13.01	125.88	13.80
**100**	80.92	11.42	91.12	22.47	89.49	12.35	115.38	13.33
**120**	71.95	10.13	79.45	13.94	95.82	10.78	104.41	15.23

Univariate Analysis of Variance

Between-Subjects Factors

		N
Sham or OVX	1	13
	2	19
Vehicle or GbE	1	16
	2	16

Tests of Between-Subjects Effects

Dependent Variable: -40 minutes

Source	Type III Sum of Squares	df	Mean Square	F	Sig.
Corrected Model	327,859[a]	3	109,286	,748	,533
Intercept	325623,299	1	325623,299	2229,568	,000
OVX	292,624	1	292,624	2,004	,168
GbE	,398	1	,398	,003	,959
OVX * GbE	31,832	1	31,832	,218	,644
Error	4089,336	28	146,048		
Total	339261,464	32			
Corrected Total	4417,194	31			

a. R Squared = ,074 (Adjusted R Squared = -,025)

Univariate Analysis of Variance

Between-Subjects Factors

		N
Sham or OVX	1	13
	2	20
Vehicle or GbE	1	17
	2	16

Tests of Between-Subjects Effects

Dependent Variable: -20 minutes

Source	Type III Sum of Squares	df	Mean Square	F	Sig.
Corrected Model	39,818[a]	3	13,273	,071	,975
Intercept	314737,308	1	314737,308	1681,810	,000
OVX	34,710	1	34,710	,185	,670
GbE	6,167	1	6,167	,033	,857
OVX * GbE	,082	1	,082	,000	,983
Error	5427,119	29	187,142		
Total	334651,441	33			
Corrected Total	5466,937	32			

a. R Squared = ,007 (Adjusted R Squared = -,095)

Univariate Analysis of Variance

Between-Subjects Factors

		N
Sham or OVX	1	12
	2	15
Vehicle or GbE	1	14
	2	13

Tests of Between-Subjects Effects

Dependent Variable: 0 minute

Source	Type III Sum of Squares	df	Mean Square	F	Sig.
Corrected Model	339,663[a]	3	113,221	,629	,603
Intercept	242494,109	1	242494,109	1347,908	,000
OVX	259,411	1	259,411	1,442	,242
GbE	55,765	1	55,765	,310	,583
OVX * GbE	7,571	1	7,571	,042	,839
Error	4137,792	23	179,904		
Total	252660,917	27			
Corrected Total	4477,454	26			

a. R Squared = ,076 (Adjusted R Squared = -,045)

Univariate Analysis of Variance

Between-Subjects Factors

		N
Sham or OVX	1	13
	2	16
Vehicle or GbE	1	15
	2	14

Tests of Between-Subjects Effects

Dependent Variable: 20 minutes

Source	Type III Sum of Squares	df	Mean Square	F	Sig.
Corrected Model	12610,191[a]	3	4203,397	3,901	,021
Intercept	320179,800	1	320179,800	297,181	,000
OVX	1195,106	1	1195,106	1,109	,302
GbE	9970,956	1	9970,956	9,255	,005
OVX * GbE	864,815	1	864,815	,803	,379
Error	26934,731	25	1077,389		
Total	357395,278	29			
Corrected Total	39544,922	28			

a. R Squared = ,319 (Adjusted R Squared = ,237)

Univariate Analysis of Variance

Between-Subjects Factors

		N
Sham or OVX	1	11
	2	16
Vehicle or GbE	1	14
	2	13

Tests of Between-Subjects Effects

Dependent Variable: 40 minutes

Source	Type III Sum of Squares	df	Mean Square	F	Sig.
Corrected Model	13015,912[a]	3	4338,637	2,876	,058
Intercept	285357,846	1	285357,846	189,141	,000
OVX	4757,400	1	4757,400	3,153	,089
GbE	2548,670	1	2548,670	1,689	,207
OVX * GbE	4236,563	1	4236,563	2,808	,107
Error	34700,284	23	1508,708		
Total	359653,251	27			
Corrected Total	47716,196	26			

a. R Squared = ,273 (Adjusted R Squared = ,178)

Univariate Analysis of Variance

Between-Subjects Factors

		N
Sham or OVX	1	13
	2	14
Vehicle or GbE	1	12
	2	15

Tests of Between-Subjects Effects

Dependent Variable: 60 minutes

Source	Type III Sum of Squares	df	Mean Square	F	Sig.
Corrected Model	20780,937[a]	3	6926,979	2,529	,082
Intercept	347265,649	1	347265,649	126,789	,000
OVX	4275,620	1	4275,620	1,561	,224
GbE	8387,521	1	8387,521	3,062	,093
OVX * GbE	4185,980	1	4185,980	1,528	,229
Error	62995,293	23	2738,926		
Total	478447,026	27			
Corrected Total	83776,230	26			

a. R Squared = ,248 (Adjusted R Squared = ,150)

Post Hoc Tests

experimental group

Multiple Comparisons

Dependent Variable: 20 minutes

Tukey HSD

(I) experimental group	(J) experimental group	Mean Difference (I-J)	Std. Error	Sig.	95% Confidence Interval Lower Bound	95% Confidence Interval Upper Bound
1	2	-26,3476	18,26137	,486	-76,5781	23,8829
	3	23,9282	16,98783	,506	-22,7993	70,6557
	4	-24,4168	16,98783	,489	-71,1443	22,3107
2	1	26,3476	18,26137	,486	-23,8829	76,5781
	3	50,2758*	17,72677	,042	1,5158	99,0359
	4	1,9308	17,72677	1,000	-46,8292	50,6909
3	1	-23,9282	16,98783	,506	-70,6557	22,7993
	2	-50,2758*	17,72677	,042	-99,0359	-1,5158
	4	-48,3450*	16,41180	,033	-93,4880	-3,2020
4	1	24,4168	16,98783	,489	-22,3107	71,1443
	2	-1,9308	17,72677	1,000	-50,6909	46,8292
	3	48,3450*	16,41180	,033	3,2020	93,4880

Based on observed means.
The error term is Mean Square(Error) = 1077,389.
*. The mean difference is significant at the 0,05 level.

Legend: 1 (Sham group); 2 (Sham+GbE group); 3 (OVX group); 4 (OVX+GbE group).

Univariate Analysis of Variance

Between-Subjects Factors

		N
Sham or OVX	1	13
	2	14
Vehicle or GbE	1	12
	2	15

Tests of Between-Subjects Effects

Dependent Variable: 80 minutes

Source	Type III Sum of Squares	df	Mean Square	F	Sig.
Corrected Model	11368,782[a]	3	3789,594	3,427	,034
Intercept	235670,718	1	235670,718	213,138	,000
OVX	2682,912	1	2682,912	2,426	,133
GbE	5851,748	1	5851,748	5,292	,031
OVX * GbE	645,277	1	645,277	,584	,453
Error	25431,533	23	1105,719		
Total	299239,993	27			
Corrected Total	36800,314	26			

a. R Squared = ,309 (Adjusted R Squared = ,219)

Univariate Analysis of Variance

Between-Subjects Factors

		N
Sham or OVX	1	12
	2	17
Vehicle or GbE	1	14
	2	15

Tests of Between-Subjects Effects

Dependent Variable: 100 minutes

Source	Type III Sum of Squares	df	Mean Square	F	Sig.
Corrected Model	5226,920[a]	3	1742,307	1,078	,376
Intercept	249473,603	1	249473,603	154,373	,000
OVX	1893,538	1	1893,538	1,172	,289
GbE	2287,120	1	2287,120	1,415	,245
OVX * GbE	432,233	1	432,233	,267	,610
Error	40401,034	25	1616,041		
Total	313389,466	29			
Corrected Total	45627,954	28			

a. R Squared = ,115 (Adjusted R Squared = ,008)

Univariate Analysis of Variance

Between-Subjects Factors

		N
Sham or OVX	1	12
	2	16
Vehicle or GbE	1	14
	2	14

Tests of Between-Subjects Effects

Dependent Variable: 120 minutes

Source	Type III Sum of Squares	df	Mean Square	F	Sig.
Corrected Model	4551,016[a]	3	1517,005	1,282	,303
Intercept	211947,494	1	211947,494	179,140	,000
OVX	4086,792	1	4086,792	3,454	,075
GbE	444,130	1	444,130	,375	,546
OVX * GbE	2,034	1	2,034	,002	,967
Error	28395,391	24	1183,141		
Total	257978,666	28			
Corrected Total	32946,408	27			

a. R Squared = ,138 (Adjusted R Squared = ,030)

Post Hoc Tests

Legend: 1 (Sham group); 2 (Sham+GbE group); 3 (OVX group); 4 (OVX+GbE group).

Tukey HSD

(I) experimental group	(J) experimental group	Mean Difference (I-J)	Std. Error	Sig.	95% Confidence Interval	
					Lower Bound	Upper Bound
1	2	-20,1262	18,49990	,700	-71,3211	31,0687
	3	-10,3969	19,47058	,950	-64,2779	43,4842
	4	-50,5351*	16,75761	,029	-96,9085	-4,1617
2	1	20,1262	18,49990	,700	-31,0687	71,3211
	3	9,7293	20,13530	,962	-45,9912	65,4498
	4	-30,4089	17,52553	,329	-78,9074	18,0896
3	1	10,3969	19,47058	,950	-43,4842	64,2779
	2	-9,7293	20,13530	,962	-65,4498	45,9912
	4	-40,1382	18,54727	,163	-91,4642	11,1877
4	1	50,5351*	16,75761	,029	4,1617	96,9085
	2	30,4089	17,52553	,329	-18,0896	78,9074
	3	40,1382	18,54727	,163	-11,1877	91,4642

Based on observed means.
The error term is Mean Square(Error) = 1105,719.
*. The mean difference is significant at the 0,05 level.

The area under the curve relating serotonin levels to time after the gavage

procedure was analyzed by two-way ANOVA adopting OVX and GbE as the main factors.

Sham group (n=6-7)		Sham+GbE group (n=5-6)		OVX group (n=5-10)		OVX+GbE group (n=7-10)	
**Mean**	**SEM**	**Mean**	**SEM**	**Mean**	**SEM**	**Mean**	**SEM**
425.7	32.10	499.3	77.26	434.0	36.03	627.5	58.69

Univariate Analysis of Variance

Between-Subjects Factors

		N
Sham or OVX	1	13
	2	18
Vehicle or GbE	1	16
	2	15

Tests of Between-Subjects Effects

Dependent Variable: AUC 5-HT levels_after gavage

Source	Type III Sum of Squares	df	Mean Square	F	Sig.
Corrected Model	224156,224[a]	3	74718,741	3,578	,027
Intercept	7420888,000	1	7420888,000	355,338	,000
OVX	35036,902	1	35036,902	1,678	,206
GbE	134171,905	1	134171,905	6,425	,017
OVX * GbE	27053,022	1	27053,022	1,295	,265
Error	563869,404	27	20884,052		
Tota[illegible]	[illegible]	[illegible]			
Corr[illegible]	[illegible]	[illegible]			

a [illegible]

Legend: 1 (Sham group); 2 (Sham+GbE group); 3 (OVX group); 4 (OVX+GbE group).

Post Hoc Tests

experimental groups

Multiple Comparisons

Dependent Variable: AUC 5-HT levels_after gavage

Tukey HSD

(I) experimental groups	(J) experimental groups	Mean Difference (I-J)	Std. Error	Sig.	95% Confidence Interval Lower Bound	95% Confidence Interval Upper Bound
1	2	-73,5833	80,39970	,797	-293,6022	146,4356
	3	-8,2778	72,82778	,999	-207,5757	191,0201
	4	-201,8000*	72,82778	,046	-401,0979	-2,5021
2	1	73,5833	80,39970	,797	-146,4356	293,6022
	3	65,3056	76,16512	,826	-143,1252	273,7363
	4	-128,2167	76,16512	,352	-336,6474	80,2140
3	1	8,2778	72,82778	,999	-191,0201	207,5757
	2	-65,3056	76,16512	,826	-273,7363	143,1252
	4	-193,5222*	68,12415	,040	-379,9483	-7,0961
4	1	201,8000*	72,82778	,046	2,5021	401,0979
	2	128,2167	76,16512	,352	-80,2140	336,6474
	3	193,5222*	68,12415	,040	7,0961	379,9483

Based on observed means.
The error term is Mean Square(Error) = 20884,052.

*. The mean difference is significant at the 0,05 level.

Western blotting data were analyzed by two-way ANOVA with OVX and GbE as main factors.

Protein (% of Sham)	Sham group (n=12-13)		Sham+GbE group (n=11-12)		OVX group (n=12-14)		OVX+GbE group (n=10-11)	
	Mean	SEM	Mean	SEM	Mean	SEM	Mean	SEM
**5-HT1A**	100.0	13.4	86.7	8.36	109.4	14.6	84.9	7.8
**5-HT1B**	100.0	7.9	90.6	4.8	88.2	8.5	98.1	7.8
**5-HT2C**	100.0	19.8	110.4	18.3	81.5	11.8	79.4	10.5
**5-HTT**	100.0	18.7	97.9	17.7	94.8	13.3	39.5	3.7
**POMC**	100.0	14.5	111.6	15.4	93.7	12.9	110.5	14.1

Univariate Analysis of Variance

Between-Subjects Factors

		N
Sham or OVX	1	24
	2	25
Vehicle or GbE	1	27
	2	22

Tests of Between-Subjects Effects

Dependent Variable: 5-HT1A

Source	Type III Sum of Squares	df	Mean Square	F	Sig.
Corrected Model	5020,526[a]	3	1673,509	,928	,435
Intercept	439636,096	1	439636,096	243,745	,000
OVX	174,289	1	174,289	,097	,757
GbE	4325,244	1	4325,244	2,398	,128
OVX * GbE	379,828	1	379,828	,211	,649
Error	81165,104	45	1803,669		
Total	540669,631	49			
Corrected Total	86185,630	48			

a. R Squared = ,058 (Adjusted R Squared = -,005)

Univariate Analysis of Variance

Between-Subjects Factors

		N
Sham or OVX	1	25
	2	25
Vehicle or GbE	1	27
	2	23

Tests of Between-Subjects Effects

Dependent Variable: 5-HT1B

Source	Type III Sum of Squares	df	Mean Square	F	Sig.
Corrected Model	1258,550[a]	3	419,517	,589	,625
Intercept	440176,498	1	440176,498	618,164	,000
OVX	57,536	1	57,536	,081	,777
GbE	,495	1	,495	,001	,979
OVX * GbE	1150,798	1	1150,798	1,616	,210
Error	32755,271	46	712,071		
Total	475870,222	50			
Corrected Total	34013,820	49			

a. R Squared = ,037 (Adjusted R Squared = -,026)

Univariate Analysis of Variance

Between-Subjects Factors

		N
Sham or OVX	1	30
	2	27
Vehicle or GbE	1	29
	2	28

Tests of Between-Subjects Effects

Dependent Variable: 5-HT2C

Source	Type III Sum of Squares	df	Mean Square	F	Sig.
Corrected Model	9558,620[a]	3	3186,207	,865	,465
Intercept	489426,106	1	489426,106	132,906	,000
OVX	8730,772	1	8730,772	2,371	,130
GbE	244,251	1	244,251	,066	,798
OVX * GbE	563,368	1	563,368	,153	,697
Error	195171,824	53	3682,487		
Total	703001,295	57			
Corrected Total	204730,444	56			

a. R Squared = ,047 (Adjusted R Squared = -,007)

Univariate Analysis of Variance

Between-Subjects Factors

		N
Sham or OVX	1	23
	2	23
Vehicle or GbE	1	23
	2	23

Tests of Between-Subjects Effects

Dependent Variable: POMC

Source	Type III Sum of Squares	df	Mean Square	F	Sig.
Corrected Model	2609,384[a]	3	869,795	,371	,774
Intercept	495935,705	1	495935,705	211,687	,000
OVX	160,037	1	160,037	,068	,795
GbE	2313,995	1	2313,995	,988	,326
OVX * GbE	77,650	1	77,650	,033	,856
Error	98396,746	42	2342,780		
Total	597340,616	46			
Corrected Total	101006,130	45			

a. R Squared = ,026 (Adjusted R Squared = -,044)

Univariate Analysis of Variance

Between-Subjects Factors

		N
Sham or OVX	1	24
	2	22
Vehicle or GbE	1	24
	2	22

Tests of Between-Subjects Effects

Dependent Variable: 5-HTT

Source	Type III Sum of Squares	df	Mean Square	F	Sig.
Corrected Model	26535,050[a]	3	8845,017	3,321	,029
Intercept	315317,280	1	315317,280	118,374	,000
OVX	11578,360	1	11578,360	4,347	,043
GbE	9382,029	1	9382,029	3,522	,068
OVX * GbE	8077,890	1	8077,890	3,033	,089
Error	111876,920	42	2663,736		
Total	470326,912	46			
Corrected Total	138411,969	45			

a. R Squared = ,192 (Adjusted R Squared = ,134)

Post Hoc Tests

experimental groups

Multiple Comparisons

Dependent Variable: 5-HTT

Tukey HSD

(I) experimental groups	(J) experimental groups	Mean Difference (I-J)	Std. Error	Sig.	95% Confidence Interval Lower Bound	Upper Bound
1	2	2,0658	21,07026	1,000	-54,2962	58,4279
	3	5,2433	21,07026	,995	-51,1187	61,6054
	4	60,4812*	22,09868	,043	1,3681	119,5942
2	1	-2,0658	21,07026	1,000	-58,4279	54,2962
	3	3,1775	21,07026	,999	-53,1846	59,5396
	4	58,4153	22,09868	,054	-,6977	117,5284
3	1	-5,2433	21,07026	,995	-61,6054	51,1187
	2	-3,1775	21,07026	,999	-59,5396	53,1846
	4	55,2378	22,09868	,075	-3,8752	114,3509
4	1	-60,4812*	22,09868	,043	-119,5942	-1,3681
	2	-58,4153	22,09868	,054	-117,5284	,6977
	3	-55,2378	22,09868	,075	-114,3509	3,8752

Based on observed means.
The error term is Mean Square(Error) = 2663,736.

*. The mean difference is significant at the 0,05 level.

Legend: 1 (Sham group); 2 (Sham+GbE group); 3 (OVX group); 4 (OVX+GbE group).
